# EEG-based Signatures of Schizophrenia, Depression, and Aberrant Aging: A Supervised Machine Learning Investigation

**DOI:** 10.1093/schbul/sbae150

**Published:** 2024-09-09

**Authors:** Elif Sarisik, David Popovic, Daniel Keeser, Adyasha Khuntia, Kolja Schiltz, Peter Falkai, Oliver Pogarell, Nikolaos Koutsouleris

**Affiliations:** Max Planck Fellow Group Precision Psychiatry, Max Planck Institute of Psychiatry, Munich, Germany; Department of Psychiatry and Psychotherapy, LMU University Hospital, LMU Munich, Munich, Germany; International Max Planck Research School for Translational Psychiatry (IMPRS-TP), Munich, Germany; Max Planck Fellow Group Precision Psychiatry, Max Planck Institute of Psychiatry, Munich, Germany; Department of Psychiatry and Psychotherapy, LMU University Hospital, LMU Munich, Munich, Germany; International Max Planck Research School for Translational Psychiatry (IMPRS-TP), Munich, Germany; German Center for Mental Health (DZPG), Partner Site Munich, Munich, Germany; Department of Psychiatry and Psychotherapy, LMU University Hospital, LMU Munich, Munich, Germany; German Center for Mental Health (DZPG), Partner Site Munich, Munich, Germany; NeuroImaging Core Unit Munich (NICUM), LMU University Hospital, LMU Munich, Munich, Germany; Munich Center for Neurosciences, LMU Munich, Munich, Germany; Department of Psychiatry and Psychotherapy, LMU University Hospital, LMU Munich, Munich, Germany; International Max Planck Research School for Translational Psychiatry (IMPRS-TP), Munich, Germany; Max Planck Fellow Group Precision Psychiatry, Max Planck Institute of Psychiatry, Munich, Germany; Max Planck Fellow Group Precision Psychiatry, Max Planck Institute of Psychiatry, Munich, Germany; Department of Psychiatry and Psychotherapy, LMU University Hospital, LMU Munich, Munich, Germany; German Center for Mental Health (DZPG), Partner Site Munich, Munich, Germany; Max Planck Fellow Group Precision Psychiatry, Max Planck Institute of Psychiatry, Munich, Germany; Max Planck Fellow Group Precision Psychiatry, Max Planck Institute of Psychiatry, Munich, Germany; Department of Psychiatry and Psychotherapy, LMU University Hospital, LMU Munich, Munich, Germany; German Center for Mental Health (DZPG), Partner Site Munich, Munich, Germany; Munich Center for Neurosciences, LMU Munich, Munich, Germany; Institute of Psychiatry, Psychology and Neuroscience, King’s College, London, UK

**Keywords:** psychosis spectrum disorders, affective disorders, support vector machine, precision psychiatry, electrophysiology, early intervention

## Abstract

**Background:**

Electroencephalography (EEG) is a noninvasive, cost-effective, and robust tool, which directly measures in vivo neuronal mass activity with high temporal resolution. Combined with state-of-the-art machine learning (ML) techniques, EEG recordings could potentially yield in silico biomarkers of severe mental disorders.

**Hypothesis:**

Pathological and physiological aging processes influence the electrophysiological signatures of schizophrenia (SCZ) and major depressive disorder (MDD).

**Study Design:**

From a single-center cohort (*N* = 735, 51.6% male) comprising healthy control individuals (HC, *N* = 245) and inpatients suffering from SCZ (*N* = 250) or MDD (*N* = 240), we acquired resting-state 19 channel-EEG recordings. Using repeated nested cross-validation, support vector machine models were trained to (1) classify patients with SCZ or MDD and HC individuals and (2) predict age in HC individuals. The age model was applied to patient groups to calculate Electrophysiological Age Gap Estimation (EphysAGE) as the difference between predicted and chronological age. The links between EphysAGE, diagnosis, and medication were then further explored.

**Study Results:**

The classification models robustly discriminated SCZ from HC (balanced accuracy, BAC = 72.7%, *P* < .001), MDD from HC (BAC = 67.0%, *P* < .001), and SCZ from MDD individuals (BAC = 63.2%, *P* < .001). Notably, central alpha (8–11 Hz) power decrease was the most consistently predictive feature for SCZ and MDD. Higher EphysAGE was associated with an increased likelihood of being misclassified as SCZ in HC and MDD (ρ_HC_ = 0.23, *P* < .001; ρ_MDD_ = 0.17, *P* = .01).

**Conclusions:**

ML models can extract electrophysiological signatures of MDD and SCZ for potential clinical use. However, the impact of aging processes on diagnostic separability calls for timely application of such models, possibly in early recognition settings.

## Introduction

Ever since Hans Berger tried to understand “psychic energy” in 1929 electroencephalography (EEG) has been used to measure mass neuronal activity.^1,2^ Over the years, both distinct and common EEG patterns for severe mental illnesses (SMIs) were established. Regarding EEG-based quantitative frequency band analysis of resting-state recordings, the increased delta (1–4 Hz) and theta (4–8 Hz) activity, which are theorized as an indicator of generalized slowing of the EEG activity, were found in both schizophrenia (SCZ) and major depressive disorder (MDD)^3–5^ and linked to dysfunctional cognitive processing.^6^ Furthermore, delta waves constitute the predominant slow rhythms in states of sleep and anesthesia which are unconscious states in a broader term^7,8^ whereas the theta band power is involved in working memory, sensory stimuli perception, and attentional control.^9,10^ SCZ patients showed abnormalities in the dominant alpha frequency (8–12 Hz) power indicative of “hypofrontality,” a state of diminished cerebral blood flow in the frontal cortex^5,11–13^ and in the gamma frequency band (30–100 Hz)^14^ which is involved in neuronal synchronization in both local and large scale neuronal networks underlying a large range of perceptual and higher order cognitive functions typically impaired in SCZ.^15–17^ Yet, patients with MDD display power changes neither in alpha activity nor beta (12–30 Hz) activity when compared to healthy control (HC) individuals.^5^ However, distinct patterns emerge in MDD, characterized by greater alpha activity in frontal regions of the left hemisphere relative to the right hemisphere, a phenomenon known as alpha asymmetry.^18^ This alpha asymmetry has been suggested to stem from disrupted emotional processing in MDD.^19^ Regarding connectivity, diverging results were found, with some studies reporting increased EEG connectivity in SMI, while others found decreased connectivity.^20,21^

Recent advances in machine learning (ML) and increased computational power have led to renewed interest in EEG techniques as a powerful and cost-effective tool to investigate neurobiological patterns of SMI offering the potential of direct, neuronal biomarkers.^22,23^ ML allows to capture complex and distributed patterns of psychiatric disorders^24,25^ and has already been successfully employed to detect SMI based on electrophysiological data.^26–30^ EEG-based models identified SCZ patients with accuracies above 71%,^31–35^ while MDD patients were detected with accuracies up to 89%.^31,36–38^ Regarding differential diagnosis between SCZ and MDD, ML models reached accuracies around 60%.^31,39,40^ However, there is a strong possibility that these high accuracies may be the result of models being overfitted on small samples.^41^ Therefore, strict cross-validation schemes, validation analyses, and thorough assessments of generalizability are needed to examine the translational robustness of such models. Additionally, exploring the underlying neural mechanisms driving these diagnostic patterns could offer valuable insights into the pathophysiology of SMIs and guide the development of more targeted interventions and treatments.

One promising measure of dysfunctional brain development is the “Brain Age Gap Estimation” (BrainAGE). BrainAGE uses supervised ML to build normative aging models, which are first trained to predict age in HC individuals and then applied to patient cohorts yielding a neurobiologically predicted age.^42^ BrainAGE is the difference in years between that predicted age and the chronological age, that is, an individualized metric to quantify the acceleration or deceleration of brain structural aging.^43^ So far, evidence for increased BrainAGE has been found across SMIs.^44^ First-episode psychosis patients show subtler deviations between +1.17 and +3.39 years,^45–49^ while SCZ patients show increased BrainAGE ranging from +2.56 to +9.00 years.^43,50–52^ In depression, effects are less pronounced, with some studies reporting non-significant results^53,54^ and others mentioning BrainAGE scores of up to +4 years.^48,54–56^ However, it remains unclear if similar electrophysiological aging processes occur. To date, few studies have explored the potential of EEG in predicting age-related disease processes and produced preliminary models that explain 33%–69% of age variance in HC.^57–60^ These changes include variance in delta and theta frequency band powers, reduced power in alpha frequency band, and increased beta band power in older individuals.^61,62^

Therefore, we employed supervised ML to explore electrophysiological patterns of SMI by (1) building and validating (differential) diagnostic classifiers separating HC, SCZ, and MDD patients based on resting-state EEG recordings, (2) constructing a novel, electrophysiological measure of brain aging estimate in SMI, that is, Electrophysiological Age Gap Estimation (EphysAGE), and (3) investigating the impact of EphysAGE on (differential) diagnostic separability. There are shared signatures between healthy aging and SMI patterns such as the decreased alpha band power as well as distinct differences in the delta and theta waves. Thus, we hypothesize a complex interplay between age-related changes and the neural signatures of SCZ and MDD to be extractable from EEG recordings by using advanced mathematical modeling, which might otherwise be obscured, or masked if lower-level univariate statistics would be employed.

## Methods and Materials

### Study Participants

The data were collected in the Department of Psychiatry and Psychotherapy of the LMU University Hospital, LMU Munich, between 2012 and 2018. A total of 735 participants were included, comprising 250 individuals, who met the diagnostic criteria for SCZ and 240 individuals for MDD according to ICD-10^63^ as well as 245 HC individuals (Supplementary table 1). The EEG recordings were performed in the clinical setting either as a routine diagnostic measure or as a part of electrophysiological studies within separate research protocols (Supplementary Methods, Supplementary table 2). All participants that are included in the studies provided written consent prior to single-site study inclusion. The study was approved by the local ethics committee of LMU (Reference Number: 22-0771). The study adhered to the ethical principles outlined by the pertinent national and institutional boards for human experimentation and followed the 2008 revised version of the Declaration of Helsinki.^64^

### Electrophysiological Data Acquisition

#### Electroencephalographic Recordings

EEG recordings of each participant were acquired in eyes-closed condition for 10 min using an electrode cap with 19 electrodes, and an additional electrooculogram channel to record eye movements placed according to the 10–20 system.^65^ A Neuroscan Synamps apparatus was used for the recordings in the Department of Psychiatry and Psychotherapy, University Hospital of the Ludwig-Maximilians-University. The sampling frequency at recording was 1000 Hz, which was downsampled to 250 Hz before further analysis steps. Electrode skin impedance was always less than 5 kΩ. All sensor electrodes were referenced to the channel Cz.

#### Data Preprocessing

The software BrainVision Analyzer (BrainVision Analyzer, Version 2.2.2, Brain Products GmbH) was used for bandpass filtering between 1 and 70 Hz, and notch filtering for 50 Hz line noise of the recordings, and all sensor electrodes were re-referenced to the average reference. The absolute values were calculated for each recording. The technicality and muscle artifacts as well as eye movement artifacts were visually inspected and cleared from the data by the trained psychiatrists and psychologists with expertise in EEG in our clinic. At least 60 s of artifact-free data segments were selected from every recording to enter the analysis pipeline.

#### Feature Extraction

Data analysis was performed with Brainstorm version 3.220503^66^ (freely available at http://neuroimage.usc.edu/brainstorm). We calculated the power spectrum density to produce frequency domain features.^67^ To better capture the nonstationarities in the oscillatory activity, Welch transformation was applied between 1 and 70 Hz with a step of 1 Hz using a window of one second with 50% overlap using fast Fourier transform defaults for every channel.^68^ This procedure yielded 1330 power spectrum density features for each participant. Furthermore, we computed NxN Pearson’s correlation coefficients between all available EEG channels amounting to 171 connectivity features per individual after eliminating the auto-correlations and doubled measurements.^69^ A total of 1501 features entered the ML pipeline.

### ML Analysis

The open-source ML platform NeuroMiner (version 1.1; http://proniapredictors.eu/neurominer/index.html) was used to train support vector machine (SVM) models with a linear kernel on both EEG modalities combined through stacking (power spectrum density, connectivity). To enhance the models’ generalizability and prevent overfitting, we adopted a pooled nested cross-validation strategy with 10-fold and 10 permutations on both the CV1 and CV2 levels. This strategy completely insulated the model training from the model testing process by performing hyperparameter optimization at the CV1 level, while validating model performance exclusively at the CV2 level.^70–73^ First, we built two SVM classification models to distinguish SCZ patients from HC individuals (SCZ classification model) and MDD patients from HC individuals (MDD classification model). Second, we built an SVM differential diagnostic model separating SCZ from MDD patients. Third, we compared our diagnostic and differential diagnostic performances to multigroup models that were trained in two different repeated nested cross-validation schemes (One-vs-all and one-vs-one, Supplementary Material). In the preprocessing pipeline of all three classification models, beta coefficients of linear associations between age and power spectrum density or connectivity features were computed in the CV1 training data of the HC group using partial correlation analysis. These coefficients were subsequently employed to correct the respective data of both patient and HC samples in the CV1 training, CV1 testing, and CV2 validation partitions (Supplementary Material).^74,75^ Third, we used support vector regression (SVR) to train and cross-validate an EphysAGE model, that is, a normative age-prediction model in the HC population (Supplementary Material). When training the model, we used explained variance (R^2^) as the optimization criterion. EphysAGE was then calculated as the difference between the predicted age from the EphysAGE model and the chronological age. The EphysAGE model was then applied to the SCZ and MDD populations to estimate their electrophysiological age.

#### Medication Effects

To decide whether to correct the EEG features for medication effects, the chlorpromazine equivalence (CPZ) dosage for antipsychotic medications^76–78^ and fluoxetine (FLUOX) dosage equivalence for antidepressant medication^79^ were calculated. We investigated associations between medication dose and the EphysAGE scores extracted from the regression model by means of Pearson’s linear correlation coefficient.^80^ Additionally, we conducted two supplementary SVM classification analyses classifying the medicated and nonmedicated patients separately for SCZ and MDD.

#### Classification Models

Balanced accuracy (BAC), defined as the mean of sensitivity and specificity, was used as the optimization criterion for the three SVM classification models. We calculated the rank-transformed decision scores^81^ produced by the three classifiers as the transformed distance between the position of the given individual in the linear kernel space and the optimally separating hyperplane. Rank transformation of decision scores was performed to enhance the interpretability and facilitate between-classifier comparisons. Specifically, a percentile rank-transformed score of 80% indicates that the subject’s score exceeds that of 79.9% of the population under study, placing the subject in the top 20% of the distribution. Hence, higher positive rank-transformed decision scores reflect increased likeness of the positive label and vice versa for the negative label. We also assessed residual age effects on classifier performance across five age bins (16–25, 26–35, 36–45, 46–55, and 56–65 years). The significance of the association between predicted labels and group membership of the models in each age bin was evaluated using the χ^2^ test.^80^ Furthermore, to assess model specificity a cross-diagnostic model application approach was implemented by applying the trained SCZ Model to MDD patients’ data and vice versa. Cross-over model performance was calculated as the percentage of individuals from the other patient group being case labeled. Finally, we used Kruskal–Wallis’ H Test^82^ and Dunn–Sidak’s post hoc comparison test^83^ (HC as reference group) to evaluate rank-transformed decision scores for group-level differences. Statistical significance was determined at α = .05.

#### EphysAGE Model

Using explained variance (*R*^2^) as the optimization criterion for the SVM regression model, we first predicted age in HC and then applied this normative model to the data of patient groups. Regression models often exhibit increased prediction errors toward the extremes of the label distribution. This phenomenon can be corrected by (1) calculating the slope between labels and prediction errors using a reference dataset and (2) applying the correction parameters on the predictions of a target sample. Therefore, this post hoc tail offset correction is customary for BrainAGE analyses.^84^ The correction parameters were calculated in our HC sample, and then used to adjust the SCZ and MDD patients’ age predictions. Group differences in EphysAGE were assessed using the nonparametric Kruskal–Wallis H test, with Dunn–Sidak’s post hoc multiple comparison tests for individual group comparisons,^82,83^ since EphysAGE scores were not normally distributed. Finally, we explored the additional value of EphysAge in our diagnostic and differential diagnostic classification models by implementing EphysAGE as an additional modality and feature to our models (Supplementary Material).

#### Model Significance and Visualization

The significance of the SVM models was assessed by comparing their performances (BAC) against a null distribution of 1000 models trained on the same dataset with random permutations of the labels.^71,85^ In each permutation, the models were retrained within the cross-validation framework using the respective label subsets corresponding to the observed-label analyses. During each permutation, predictions were gathered from randomized models to create a permuted ensemble prediction for each CV2 subject. Consequently, a null distribution of out-of-training classification performance was established for the prediction models. To assess the significance of the observed out-of-training BAC, we computed the number of instances where the permuted out-of-training BAC equaled or exceeded the observed BAC. Model significance was determined at a *P* value threshold of <.05.

Models were visualized by back-projecting model weights to the original data space. Pattern element stability and significance of the SVM model feature weights were estimated using the measures of cross-validation ratio (CVR) and sign-based consistency, respectively.^86,87^ CVR represents the sum of the median weights from all CV1 folds, divided by the standard deviation. It is an indicator of how stable features contribute to the predictive pattern. Sign-based consistency assesses how consistently a variable was either positively or negatively weighted across the CV partitions. This method allows us to compute a *P* value for each feature in the dataset to determine whether this sign-based consistency is above the chance level. Features with a CVR ≥ |2| and a false discovery rate corrected *P* value <.05 were defined as reliable and significant predictors. Specifically, the median of the CVR for each frequency interval: delta (1–3 Hz), theta (4–7 Hz), alpha (8–11 Hz), beta (12–29 Hz), low-gamma (30–49 Hz), and high-gamma (50–70 Hz) was calculated for those features, which were deemed significant by sign-based consistency mapping. Furthermore, the association between chronological age and power spectrum density was investigated with Pearson’s correlation method (Supplementary Material). The CVR values of correlation-based EEG features were further analyzed using an undirected graph-theoretical approach.^88^ Subsequently, their contribution to the models’ predictive patterns was assessed via eigenvector centrality which is a measure of the influence of a node, for example, channels, in a network^88^ (Supplementary Material).

## Results

### Sample Characteristics

SCZ patients (mean[SD], 36.5[11.4]) were significantly younger and more predominantly male (93, 62.8% male) compared to MDD patients (44.3[14.4], *P* = 1.12 × 10^−8^, 145 (39.4% male) and HC individuals (47.0[4.5], *P* = 9.67 × 10^−16^, 121 (51.14% male). 146 out of 240 (60.8%) MDD patients and 90 out of 250 (36.0%) SCZ patients were unmedicated (Supplementary table 1).

### Medication Effects

The EphysAGE scores of medicated patients were not correlated with CPZ dosage for SCZ and FLUOX dosage for the MDD group (*ρ*_CPZ_ = −0.05, *P* = .60 and *ρ*_FLUOX_ = −0.06, *P* = .57). Additionally, the two models distinguishing medicated from nonmedicated patients with the SCZ or MDD groups did show predictive performances close to chance levels (SCZ: BAC = 55%; MDD: BAC = 52%; Supplementary Material). Therefore, we did not correct for medication effects in the differential diagnostic or age prediction analyses.

### Classification Models

**Fig. 1.**
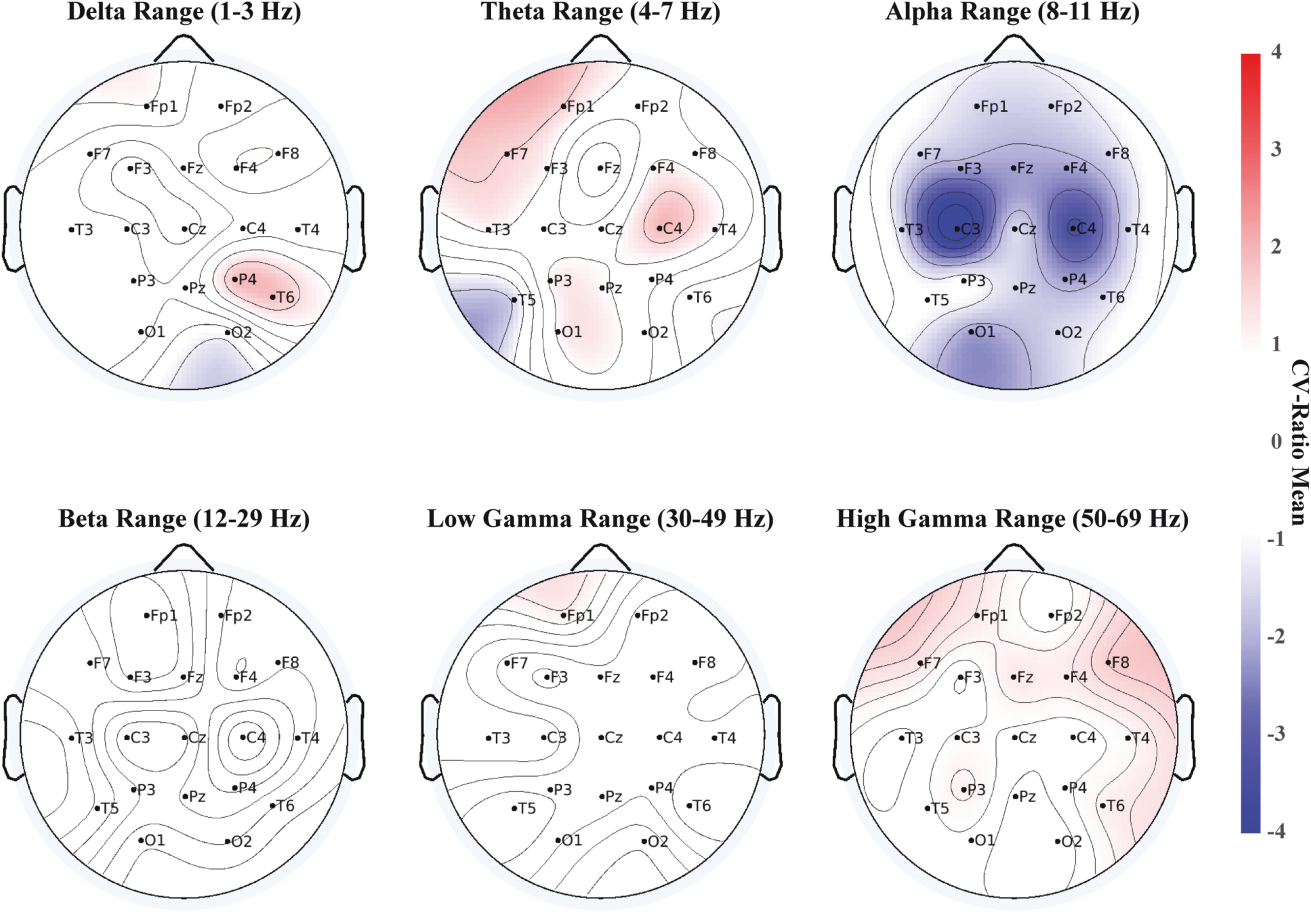
Topographical plots of overall mean of CVR in classification models—SCZ Model. The topographical representations with channel locations and names of CV ratio overall mean values in the frequency power domain for three classification models are illustrated. Frequency ranges: delta: 1–3 Hz, theta: 3–7 Hz, alpha: 8–11 Hz, beta: 12–19 Hz, low-gamma: 20–49 Hz, high-gamma: 50–70 Hz. SCZ Model: decreased alpha frequency power was predictive of SCZ likeness.

The SCZ classification model correctly identified 179 out of 250 SCZ (sensitivity, SEN = 71.6%) and 181 out of 245 HC individuals (specificity, SPEC = 73.9%), yielding a cross-validated BAC of 72.7%, a positive predictive value (PPV) of 73.7% and a negative predictive value (NPV) of 71.8% (*P*_1000_ < .001). In the SCZ model, global alpha oscillation power decrease was predictive of SCZ (figure 1). Centrality values derived from the undirected graph network peaked between the C4 and F7 followed by Fp1 electrodes.

**Fig. 2.**
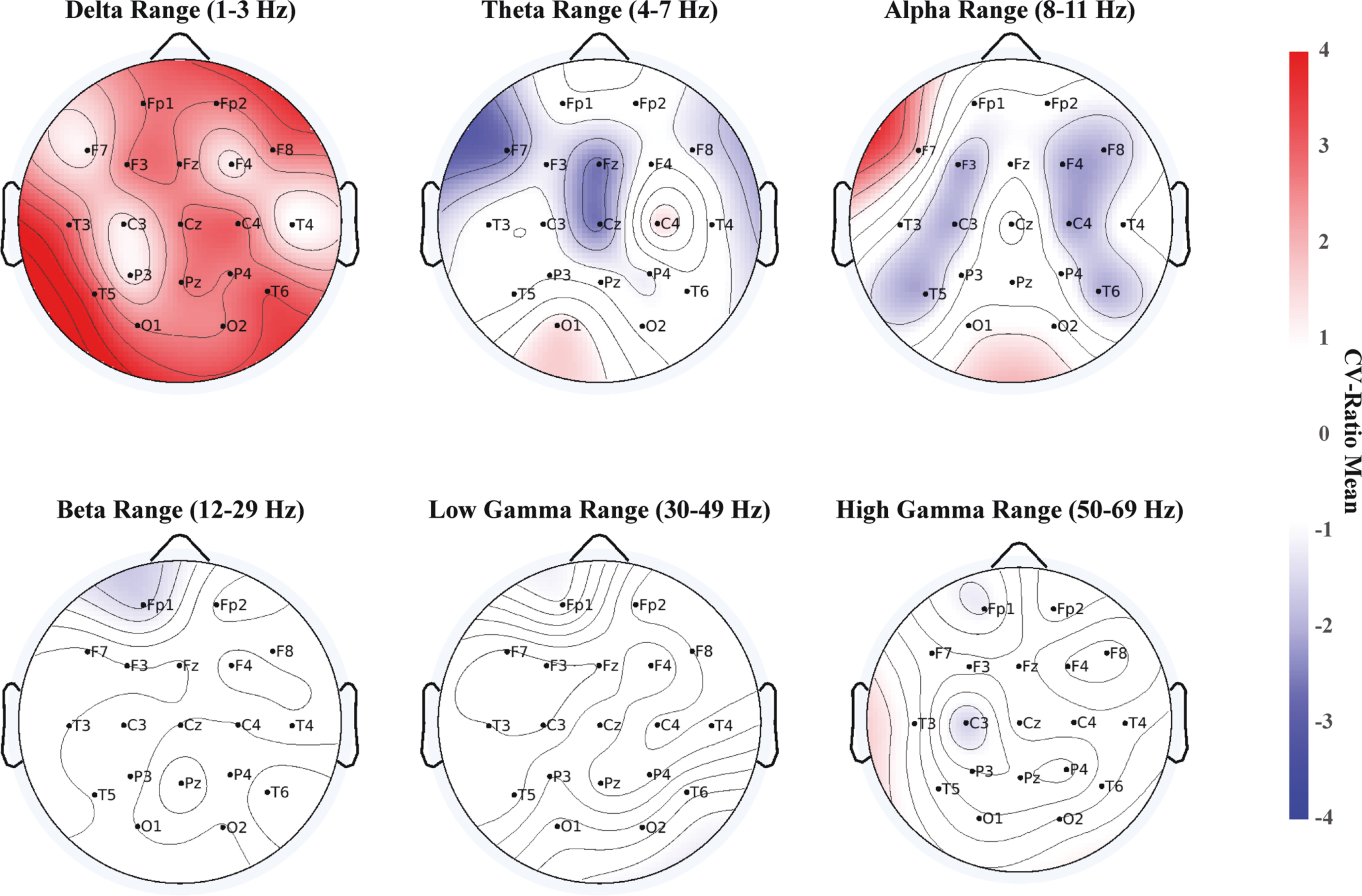
Topographical plots of overall mean of CVR in classification models—MDD Model. Overall increased delta frequency power decreased central and left temporal theta power as well as decreased central alpha power along with decreased left temporal alpha power were predictive of MDD likeness.

The MDD classification model correctly identified 163 out of 240 MDD (SEN = 67.9%) and 162 out of 245 HC individuals (SPEC = 66.1%), with a cross-validated BAC of 67%, a PPV of 66.3%, and an NPV of 67.8% (*P*_1000_ < .001). Higher global delta frequency power was predictive of MDD status, while frontal theta frequency power contributed to HC classification (figure 2). The MDD Model showed the highest centrality in central and frontal regions based on the undirected graph.

The classification performance of the SCZ and MDD Models peaked in the second youngest (26–35 years) age bin (SCZ: SEN = 85.7%, χ^2^ = 31.2, *P* = 2.35e^−6^; MDD: SEN = 80.0%, χ^2^ = 23.8, *P* = 1.06e^−6^). Both classification models performed worst in the oldest age group (56–65 years, SCZ: SEN = 42.9%, χ^2^ = 9.7, *P* = .002; MDD: SEN = 61.9%, χ^2^ = 13.7, *P* = 2.13e^−4^; table 1).

**Table 1.** Performance Metrics of the EEG-based Classification Models

	SENS (%) (95% CI)	SPEC (%) (95% CI)	BAC (%) (95% CI)	PPV (%) (95% CI)	NPV (%) (95% CI)	AUC (95% CI)	*χ* ^2^	*P*
SCZ Classification Model								
Overall (*N* = 495)	71.60 (67.91–73.32)	73.88 (70.07–76.96)	72.73 (69.71–74.42)	73.66 (70.36–75.92)	71.83 (68.76–73.30)	0.73 (0.68–0.77)	102.4	**4.54E-10**
16–25 years (*N* = 65)	84.78 (79.01–86.95)	52.63 (42.71–64.29)	68.71 (62.36–74.13)	81.25 (77.50–84.98)	58.82 (48.66–64.35)	0.69 (0.55–0.82)	9.75	**.002**
26–35 years (*N* = 127)	85.71 (80.46–88.25)	62.00 (57.16–68.21)	73.86 (70.01–76.99)	77.65 (74.94–80.42)	73.81 (66.82–77.73)	0.74 (0.65–0.82)	31.18	**2.35E + 06**
36–45 years (*N* = 118)	69.62 (64.23–74.20)	61.54 (57.37–73.94)	65.58 (62.53–72.33)	78.57 (76.32–84.38)	50.00 (46.13–56.50)	0.66 (0.55–0.76)	10.5	**.001**
46–55 years (*N* = 73)	40.63 (28.76–47.88)	73.17 (70.60–79.21)	56.90 (51.36–61.50)	54.17 (47.55–60.95)	61.22 (57.14–64.71)	0.57 (0.44–0.70)	1.55	.21
56–65 years (*N* = 106)	42.86 (28.31–63.23)	89.13 (78.99–91.40)	65.99 (57.36–73.64)	37.50 (21.78–42.82)	91.11 (88.76–93.64)	0.66 (0.50–0.82)	9.7	**.002**
MDD Classification Model								
Overall (*N* = 485)	67.92 (63.48–70.72)	66.12 (63.92–69.55)	67.02 (64.60–69.24)	66.26 (64.13–68.67)	67.78 (64.82–70.07)	0.67 (0.62–0.72)	56.2	**6.54E-14**
16–25 years (*N* = 43)	58.33 (50.20–71.31)	47.37 (38.76–56.74)	52.85 (46.53–61.97)	58.33 (52.82–66.07)	47.37 (40.05–58.33)	0.53 (0.35–0.70)	0.14	.71
26–35 years (*N* = 95)	80.00 (69.08–84.33)	70.00 (64.64–76.59)	75.00 (68.77–78.54)	70.59 (65.32–75.01)	79.55 (71.28–83.10)	0.75 (0.65–0.85)	23.81	**1.06E-06**
36–45 years (*N* = 103)	68.75 (61.62–73.39)	66.67 (58.60–71.09)	67.71 (62.50–70.85)	77.19 (72.00–79.82)	56.52 (49.42–60.46)	0.68 (0.57–0.78)	12.3	**4.53E-04**
46–55 years (*N* = 88)	68.09 (58.20–75.21)	56.10 (52.47–67.67)	62.09 (57.51–69.28)	64.00 (60.38–71.04)	60.53 (54.27–68.21)	0.62 (0.50–0.74)	5.22	**.02**
56–65 years (*N* = 136)	61.92 (56.02–67.95)	71.74 (68.45–76.34)	66.55 (64.03–70.30)	50.94 (48.01–55.54)	79.52 (77.48–82.38)	0.67 (0.56–0.77)	13.71	**2.13E-04**
Differential Diagnostic Model (SCZ vs MDD)								
Overall (*N* = 490)	71.6 (69.30–74.41)	54.17 (51.74–57.73)	62.88 (61.22–65.37)	61.94 (60.48–64.16)	64.68 (62.60–67.64)	0.63 (0.58–0.68)	33.6	**6.76E-09**
16–25 years (*N* = 70)	97.83 (94.32–100)	0 (–2.03–2.87)	48.91 (47.35–50.35)	65.22 (64.49–65.88)	0	0.49 (0.34–0.63)	0.53	.47
26–35 years (*N* = 122)	97.40 (92.04–99.05)	17.78 (12.20–24.13)	57.59 (53.61–60.09)	66.96 (64.95–68.35)	80.00 (54.87–87.02)	0.58 (0.47–0.68)	8.7	**.003**
36–45 years (*N* = 143)	70.89 (66.77–77.40)	34.38 (30.58–44.75)	52.63 (51.03–58.72)	57.14 (55.96–61.68)	48.89 (46.34–58.09)	0.53 (0.43–0.62)	0.45	.5
46–55 years (*N* = 79)	9.38 (6.50–19.58)	87.23 (81.03–92.20)	48.30 (45.50–54.16)	33.33 (23.40–56.48)	58.57 (56.99–61.81)	0.48 (0.35–0.61)	0.22	.64
56–65 years (*N* = 58)	0 (–4.16–6.98)	97.73 (92.15–100)	48.86 (45.15–52.36)	0	75.44 (74.01–76.77)	0.49 (0.31–0.66)	0.32	.57

*Note*: The performance of three classification models (SCZ, MDD, and Differential Diagnostic Models) are represented in five different 10-year apart age bins (16–25, 26–35, 36–45, 46–55, 56–65) along with their overall performances. Highest sensitivity was found in the youngest age bin for SCZ and Differential Diagnostic Model and in the 36–45 years age bin for MDD Model. In all three models, the highest BAC was achieved in the 26–36 years of age group. SEN, sensitivity; SPEC, Specificity; BAC, balanced accuracy; NPV, negative predictive value; PPV, positive predictive value; *P* value threshold = .05.

The differential diagnostic model (SCZ vs MDD) correctly detected 179 of 250 SCZ (71.6%) and 130 of 240 MDD patients (54.2%), resulting in a BAC of 62.88%, a PPV of 61.94%, and a NPV of 64.68% (*P*_1000_ < .001). Overall delta frequency power was indicative of SCZ class membership while high-gamma oscillations predicted MDD status (Supplementary figure 3). The highest centrality was observed for the P3, C4, and Fp1 electrodes.

**Fig. 3.**
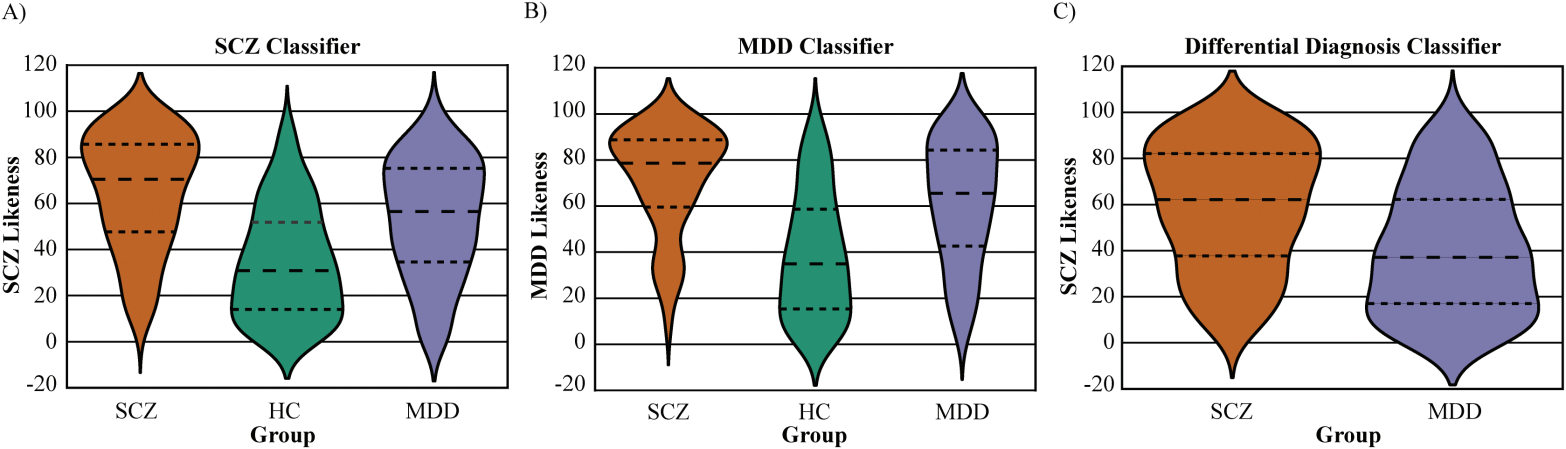
Model specificity assessment with cross-diagnostic application. Difference of the mean decision scores in different groups for all classification models. (A) In SCZ Model, all three groups’ decision scores are different from each other, and the highest decision scores are in the patients with SCZ followed by patients with MDD then the HC individuals. (B) In MDD model, again the three groups are different from each other in terms of their decision scores and the gradual change is in the same ranking as the SCZ Model. (C) In the differential diagnostic model, the mean decision scores in patients with SCZ are significantly higher than in MDD patients. All three models indicate a gradual change in the disease signature between SCZ and MDD.

The SCZ Model case labeled, that is, labeled as SCZ, 55.0% of the MDD patients. Specifically, SCZ patients had the highest rank-transformed decision scores, that is, the likelihood to be classified as SCZ (median[interquartile range (IQR)], 70.5[IQR:38.2]) followed by MDD patients (56.5[IQR:40.8]) and HC individuals (30.8[IQR:38.0]; χ^2^ = 151.6, df = 2, *P* = 1.2e-33). The MDD Model case labeled, that is, labeled as MDD, 82.10% of the SCZ individuals (figure 3). Notably, SCZ patients yielded higher rank-transformed decision scores (78.7[IQR:29.2]), that is, a higher likelihood to be classified as MDD, compared to MDD patients (65.6[IQR:42.0]), and HC individuals (34.9[IQR:43.5], χ^2^ = 160.0, df = 2, *P* = 1.8e-35).

The multigroup classifier trained in an all-vs-one repeated nested cross-validation scheme which distinguishes one group from the two other groups, performed at chance level (Supplementary figures 1 and 2). In contrast, the multigroup classifier trained in a one-vs-one cross-validation scheme performed at a similar level to the original binary classifiers (Supplementary figures 2 and 3).

Furthermore, the SVM classifiers distinguishing medicated from nonmedicated patients did not identify medicated individuals above chance level after permutation testing (*P* > .05), which supported our initial approach not to correct our models for medication effects (Supplementary Material). Additionally, the fluoxetine and CPZs were not significantly correlated with EphysAGE in medicated MDD (*P* = −.06, *P* = .57) or SCZ patients (*P* = −.05, *P* = .60).

### EphysAGE Model

The EphysAGE model predicted age in HC individuals with an explained variance of 46% (MAE = 8.7 years, T = 14.31, *P*_1000_ < .001). Central higher delta frequency as well as occipital and temporal lower high-gamma frequency power predicted higher age (Supplementary figure 4). Centrality in the EphysAGE model peaked at T5, T4, and P3 electrode regions (Supplementary table 5).

When applied to the two patient groups, the Kruskal–Wallis H test revealed significant differences between HC, MDD, and SCZ individuals (χ^2^ = 6.46, *P* = .04). Dunn–Sidak’s post hoc multiple comparison test showed no significant difference between patient groups and HC regarding their EphysAGE (SCZ vs HC, *P* = .80; MDD vs HC, *P* = .17). However, the EphysAGE of SCZ patients was significantly higher than of MDD patients (SCZ vs MDD, *P* = .04).

### Interaction Between EphysAGE and (Differential) Classification Performance

**Fig. 4.**
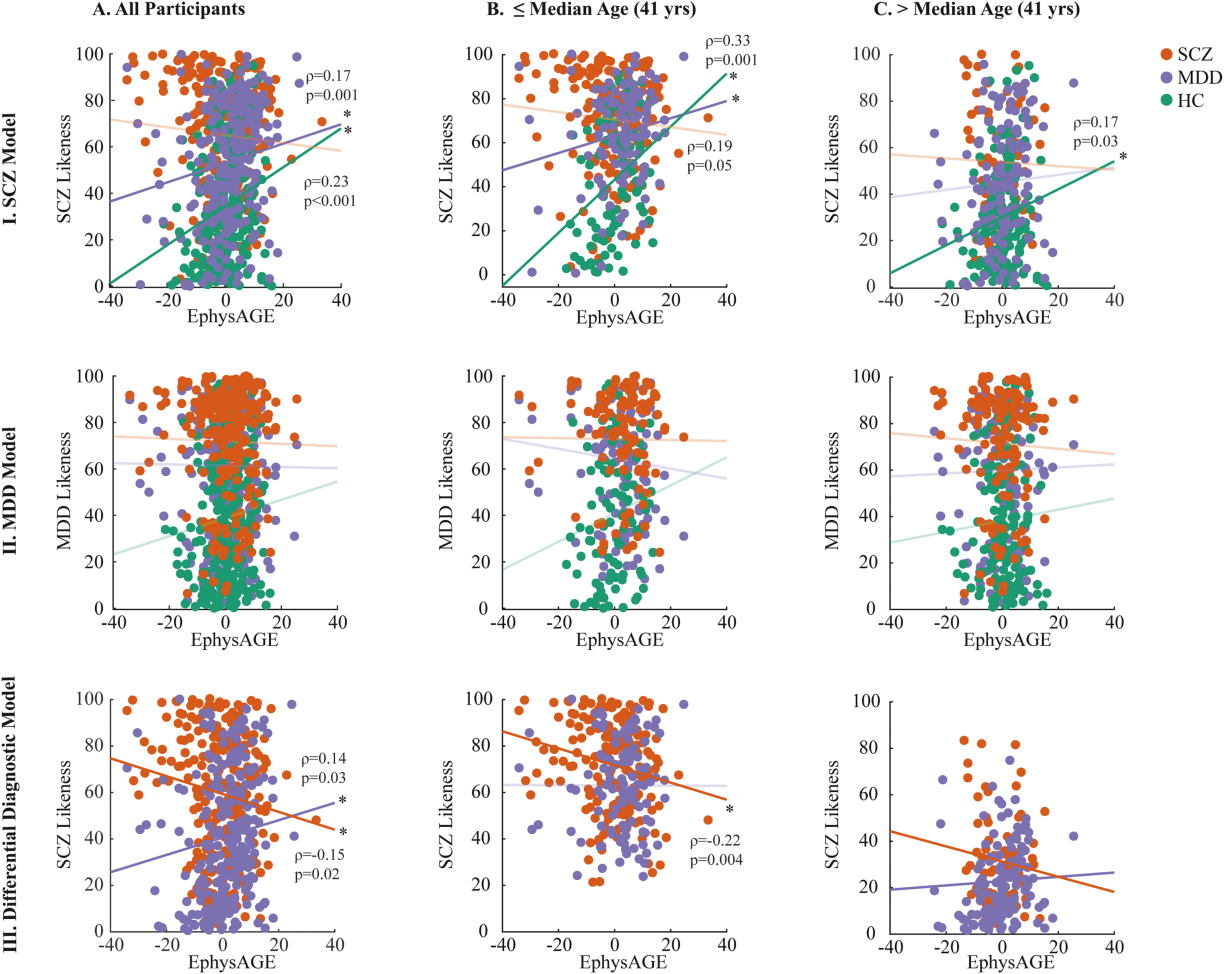
EphysAGE correlations with SMI likeness. Correlations between rank-transformed decision scores derived from the classification models and EphysAGE are investigated in younger and older age groups. In the SCZ Model, (I) EphysAGE of MDD and HC groups are positively correlated with SCZ-likeness, that is, when EphysAGE is higher in these two groups, they are more likely to be misclassified as SCZ. These correlations are more prominent in the younger age groups. (II) In the MDD Model, there is no significant correlation between the group assignment and EphysAGE in any age group. In the differential diagnostic model, (III) Higher EphysAGE in the SCZ group is correlated with misclassification as MDD and higher EphysAGE in the MDD group is correlated with misclassification as SCZ in the all participant group. The correlation between the SCZ group’s misclassification with higher EphysAGE consists in the young age group.

The EphysAGE scores of HC individuals and MDD patients were positively correlated with their rank-transformed SCZ scores indicating that their SCZ classification likelihood increased with higher EphysAGE (*ρ*_HC_ = 0.23, *P* = 3.42 × 10^−4^; ρ_MDD_ = 0.17, *P* = .01). No such correlation was found for SCZ individuals (*ρ*_SCZ_ = −0.08, *P* = .21, figure 4). Also, no significant correlations were detected between EphysAGE and rank-transformed MDD decision scores. In the differential diagnostic model, a significant correlation between EphysAGE and rank-transformed decision scores was observed found: In the SCZ group, SCZ likeness decreased with higher EphysAGE (*ρ*_SCZ_ = −0.15, *P* = .02) while it increased in the MDD sample (*ρ*_MDD_ = 0.14, *P* = .03). Finally, the models that contained EphysAGE as an additional feature performed at similar levels with the original binary classifiers. The EphysAGE was a predictive feature only when added to the differential diagnostic classifier (CVR = −2.15, Supplementary Material).

## Discussion

We used resting-state EEG and supervised ML in a large, naturalistic single-site cohort to quantify aberrant brain aging processes and assess their impact on diagnostic separability. First, we developed three EEG-based classification models that robustly discriminated SCZ and MDD patients from HC individuals, and, to a lesser degree, SCZ from MDD patients. Second, we developed an EEG-based normative age prediction model, revealing that SCZ patients had higher EphysAGE than MDD patients. Third, we showed that higher electrophysiological brain aging, that is, higher EphysAGE scores, negatively impacted the identification of MDD and HC in the SCZ classification model and the identification of both MDD and SCZ patients in the differential diagnostic model (SCZ vs MDD).

Exploration of the SCZ model revealed alpha power decrease in frontal, central, and left occipital brain regions as the most predictive features for SCZ, which is in line with previous findings.^89–91^ In these previous studies, reduced alpha power and decreased alpha coherence were correlated with negative symptoms, impairments in visual perception, and poorer cognitive performance in task-dependent conditions,^90,92–94^ thereby linking these changes to early disrupted perceptual processing in SCZ. In resting-state conditions, alpha rhythm voltage has been linked to a decrease in cortical excitability and attention.^95^. Moreover, a link has been suggested between alpha rhythm amplitude, cortical excitability, and cholinergic pathways in the forebrain.^95,96^ Thus, this pattern could represent an electrophysiological signature of SCZ, linking widespread alpha power decrease as well as dysfunctional cholinergic transmission and glutamatergic hypofunction to characteristic clinical impairments.^97,98^ Moreover, these processes are speculated as being facilitated by reduced kainate receptor density in both psychotic and affective disorders.^99^

The MDD classification model rested on overall delta power increase and alpha power decrease to separate study participants. While these findings further support the role of disrupted alpha rhythm in MDD, they do not replicate the previously reported frontal alpha asymmetry in MDD^100,101^ which was in itself rebutted in a recently published meta-analysis.^18^ Rather, a central bilateral alpha power decrease was observed in our model derived from our naturalistic sample.

Moreover, we observed classification asymmetry in our cross-diagnostic analysis, with the SCZ model case-labeling MDD individuals in 55.8% of cases, while the case-labeling of SCZ patients occurred at a much higher rate (82.1%) in the MDD Model. Hence, the SCZ Model might have captured an electrophysiological signature that is more specific to SCZ pathophysiology, while the MDD Model represents a more general SMI signature, which distinguishes MDD and SCZ as SMIs in general from HC individuals. This interpretation may be seen as consistent with the triadic system of mental illness^102^ where depressive symptoms constitute a transnosological external circle common to many different psychiatric disorders which envelopes an inner circle that contains SCZ characterized by more specific, or endogenous symptoms.^48,103^ An alternative explanation of our findings could align with the hypothesis that a subgroup of MDD is more closely aligned with SCZ in terms of a common functional brain substrate, while the rest of the MDD population exhibits a mixed brain pathology not related to SCZ.^75^ The one-vs-all multigroup classifiers performed significantly much lower than the original binary classification models. This could be attributed to the greater diversity within the “all” groups, which included combinations of HC and MDD, HC and SCZ, or MDD and SCZ, depending on the setup. On the other hand, the one-vs-one multigroup classification approach involves pairwise classifications between two distinct groups. This makes them comparable to the binary models which might, in turn, explain why they exhibited similar performance levels.

In the EphysAGE model, the spectral features most strongly predictive of older age included increased power in overall delta and frontal and temporal theta frequency intervals. Although a slow-wave power change in healthy aging was consistently reported in the literature,^10,61,62,104,105^ there is still much controversy regarding the direction of this power change. While several studies found a decrease in slow waves in EEG and magnetoencephalography with increased age,^62,106,107^ other studies reported an increase in delta and theta power correlating with older age in resting-state recordings.^10^ Notably, the alpha power which was found to decrease in healthy aging in previous studies,^10,62,108,109^ was not a significant predictor of age in our model. Consistent with previous studies, we found frontotemporal and central electrodes to be predictive of higher age.^110^ Similar to the MRI-based BrainAGE measure,^111,112^ progressive and regressive neurobiological processes such as the glucocorticoid cascading hypothesis, which suggests a feed-forward mechanism between the psychosocial daily stressors and both aging and psychotic experiences^113,114^ might also underlie these electrophysiological patterns. Therefore, pathological accelerated aging is not only detectable in structural brain patterns but can in fact also be observed in changes in neural activity as measured by EphysAGE.

Furthermore, the correlation between EphysAGE and classification likelihood offers additional insight into the link between accelerated aging, disease-related electrophysiological aberrations, and diagnostic separability. The positive correlation between EphysAGE in MDD and HC individuals and the likelihood of being case labeled as SCZ could be reflective of a pathophysiological proximity between MDD patients with accelerated brain aging and SCZ patients.^48,115,116^ Moreover, the difference in EphysAGE between individuals with MDD and SCZ could further substantiate this argument. For SCZ individuals, the inverse association between EphysAGE and the likelihood of being correctly classified in the differential diagnostic model might indicate that the electrophysiological disease signature of SCZ becomes “diluted” as the disease progresses and EphysAGE increases. This could be speculatively interpreted as a “multilayered aging” process where the electrophysiological disease signatures become increasingly blurred and less specific, therefore being increasingly masked by diffuse aging and degeneration patterns^43,48^ caused, for example, by the somatic sequelae of poor lifestyles, and/or social deprivation effects. Further support for this hypothesis is derived from our finding that with increasing age, the sensitivity of the SCZ and MDD classification models decreases, while their specificity increases. Hence, in contrast to BrainAGE findings, the models become less accurate in identifying the disorders with higher age in EEG recordings, which, again, could be attributed to an increased “dilution” of discriminative neural signals. Indeed, previous studies found that the structural brain signature of SCZ becomes more distributed and diffuse as the disease progresses, making it less distinct.^117^ Therefore, BrainAGE, a structural signature, differs fundamentally from EphysAGE, an electrophysiological signature, as BrainAGE appears more pronounced and predictive as age increases.^118^ EphysAGE, on the other hand, becomes more diffuse and less predictive. This could be attributed to counteracting processes between physiological aging and disease effects of SCZ and MDD, such as inverse directionality in high-gamma oscillations power and absence or presence of theta and delta frequency powers patterns. Therefore, younger patients in earlier disease stages would carry more specific electrophysiological disease signals, which are not yet overly influenced by physiological and pathological aging processes, making EEG-based biomarkers most well-suited for early recognition and intervention purposes. Furthermore, our results suggest that EphysAGE may be most effective when used in conjunction with other features rather than as the sole predictor, especially in the differential diagnosis.

Beyond these findings, several limitations should be noted. First, the lack of a deeper clinical phenotyping of our sample did not allow for a more in-depth investigation of possible crosslinks and interactions between EphysAGE and other markers of disease progression. Thus, more deeply phenotyped samples would enable more fine-grained investigations into how prediction performance, EphysAGE, and feature importances may be influenced by the age of onset and the duration of the disease, the outcome of the treatment as well as the effect of the severity of different symptoms. Through these analyses, researchers could gain a more comprehensive understanding of how different disease facets impact on neural activity. Such investigations hold the potential to unveil novel biomarkers, enhance predictive models, and ultimately advance diagnostic and treatment approaches for individuals impacted by SMI. Furthermore, the substantial chronological age gap between SCZ and HC individuals might have negatively impacted the EphysAGE model since the accelerated brain aging in SCZ individuals might have brought them “biologically closer” to the already older HC population, even though the classification models were corrected for age in the classification models. Moreover, only the eyes-open condition of the resting-state EEG recordings was included in the current work (Supplementary Material). Lastly, external validation approaches should be employed to assess the generalizability of our models in independent samples.

## Conclusion

In summary, our findings indicate that ML techniques applied to routine EEG data can generate diagnostic models. These models should be translated into clinical practice in early disease stages where the impact of physiological and pathological aging processes is still limited, and the electrophysiological brain signatures provide higher discriminative power. A timely and targeted application of such EEG-based models could lead to noninvasive, cost-effective biomarkers of neural activity in severe mental disorders.

## Supplementary Material

Supplementary material is available at https://academic.oup.com/schizophreniabulletin.

sbae150_suppl_Supplementary_Tables_S1-S5_Figures_S1-S4
