# Supplementary material for: EEG-based Signatures of Schizophrenia, Depression, and Aberrant Aging: A Supervised Machine Learning Investigation
Source: Schizophr Bull. 2024 Sep 9;51(3):804–17. doi: 10.1093/schbul/sbae150 (PMC12061654; doi:10.1093/schbul/sbae150)
Supplement: sbae150_suppl_Supplementary_Tables_S1-S5_Figures_S1-S4 [file sbae150_suppl_supplementary_tables_s1-s5_figures_s1-s4.docx]

**EEG-based signatures of schizophrenia, depression, and aberrant aging: a supervised machine learning investigation**

Elif Sarisik (ORCID-ID:0000-0003-3736-2462)^1,2,3^*_,_ David Popovic (ORCID-ID:0000-0002-2367-9437)^1,2,3,4^*, Daniel Keeser (ORCID-ID:0000-0002-0244-1024) ^2,4,5,6^, Adyasha Khuntia (ORCID-ID:0009-0007-9297-5997)^2,3^, Kolja Schiltz (ORCID-ID:0000-0003-3788-3241) ^1^, Peter Falkai (ORCID-ID:0000-0003-2873-8667) ^1,2,4^, Oliver Pogarell (ORCID ID:0000-0001-6455-4190) ^1**^, Nikolaos Koutsouleris (ORCID-ID:0000-0001-6825-6262)^1,2,4,6**, CA^

**Running Title:** EEG signatures of severe mental illness and aging

1 Max Planck Institute of Psychiatry, Munich, Germany

2 Department of Psychiatry and Psychotherapy, LMU University Hospital, LMU Munich, Munich, Germany

3 International Max Planck Research School for Translational Psychiatry (IMPRS-TP), Munich, Germany

4 German Center for Mental Health (DZPG), partner site Munich

5 NeuroImaging Core Unit Munich (NICUM), University Hospital LMU, Munich, Germany

6 Munich Center for Neurosciences, LMU, Munich, Germany

7 Institute of Psychiatry, Psychology and Neuroscience, King’s College, London, UK

* Shared first authors

** Shared last authors

***Supplementary Material***

# **Supplementary Methods**

## **Participant Source Studies and Demographic Information**

The participant demographics and source studies for participant extraction are summarized in Table S1 and Table S2. The study named "Rebox" explores the differential effects of serotonergic and norepinephrinergic antidepressants on individuals. In the "Remergil" study, researchers investigate the neurobiological effects of mirtazapine. The "Katamnese Depression" study focuses on the long-term clinical and neurobiological characterization of patients with depression. For "Katamnese Schizophrenie," the research involves the long-term examination of patients with schizophrenia. The "TMS" study aims to conduct a neurophysiological investigation of patients both before and during treatment with transcranial magnetic stimulation (TMS). In the "Genetik" study, researchers aim to provide a clinical, neurophysiological, and genetic characterization of a large community cohort. The "MRP300" study involves the neurophysiological and neuroimaging characterization of patients with schizophrenia. The "Akut" study investigates the neurophysiological parameters of patients with schizophrenia both before and during treatment. The "Basis-Schizophrenie" study focuses on the clinical and neurobiological characterization of patients with schizophrenia. Finally, the "DynSpect" study aims to investigate patients with schizophrenia at baseline and under dopaminergic challenge.

## **Machine Learning Pipeline**

The power spectrum density data modality was mean-standardized and connectivity data modality was scaled between 0 and 1. For the MDD and SCZ model training, a preprocessing pipeline was implemented, in which standardization and scaling steps were conducted first and then covariate correction for age effects was performed. To correct for age-related effects, it is crucial to distinguish them from disorder-related changes. Therefore, before the SVM classification model, we implemented an additional General Linear Model (GLM) exclusively on healthy control individuals (see also Dukart et al. 2011, https://pubmed.ncbi.nlm.nih.gov/21829449/). GLMs are calculated for all EEG data features yc separately. The matrix Xc includes a constant and age as its columns and only the group of healthy control individuals are used to determine the regression coefficients ß, consisting of ß0 for the constant ßc for age-related changes at each feature. The GLM is represented by the equation:

*y_C_ = X_C_ß + e_C_*

Solving this equation for the least square estimates of ß satisfies the normal equations:

$x_{C}^{T}x_{C}\beta=x_{C}^{T}$*y_C_*

Solving this system of linear equations for ß results in:

$$\beta=\left( x_{C}^{T}x_{C} \right)^{-1}x_{C}^{T}y_{C}$$

To obtain age-corrected EEG features yCOR the calculated age regression coefficients ßC are applied to the corresponding features in EEG data of both healthy control individuals and patients with SCZ and MDD. The residual amount explained by each subject’s individual age XA is removed from the observed EEG feature value yA at each feature using the determined coefficient ßC.

*Y_COR_ = y_A_ - ß_C_X_A_*

It is crucial to use only healthy control individuals to determine the regression coefficient, as patients with SCZ and MDD might exhibit different patterns of brain activity in EEG data. Thus, removing age-related effects using regression coefficients determined in the patient groups might inadvertently remove disease-related changes due to their interaction with age.

For the differential diagnostic model, this covariate correction step was slightly adapted since the cross-validation structure contained only MDD and SCZ individuals. Therefore, the beta coefficients were computed in the HC sample outside of NeuroMiner and then imported to the preprocessing pipeline of the differential diagnostic model to correct the EEG features of the patient groups in the same way as done in the SCZ and MDD classification model. For all models, this age correction step was separately applied to power spectrum density and connectivity features.

In the classification models L1-regularized linear LIBSVM version 3.1.2 was used. The 11 selected C-parameters were started from 0.015625 to 2. For all classifier models, two SVMs were trained based on above mentioned two different data modalities, and another stacking model was trained on the decision scores from previous SVMs scaled between 0 to 1. LIBSVM version 3.1.2 with instance weighting support was also used in the regression model with a regressor type of epsilon-SVR (0.1). The 7 slack parameters were selected between 0.015625 and 1, whereas 6 epsilon-SVR parameters were used between 0.05 and 2.

**Interplay of Medication and Age Effects**

To assess medication-depended age effects, we split the SCZ (36.45 ±11.40 years) and MDD (44.29 ±14.44 years) group based on their median age into two groups (median split). We then compared the older and younger groups within each patient group in terms of their correlation between the medication dosage profile and EphysAGE. Furthermore, we investigated if there is any significant group difference between the younger and older patients in terms of their rank transformed decision scores based on the medicated vs. non-medicated classification models with analysis of variance^3^.

**Multiclass Support Vector Machine Model**

In addition to the three binary classification models, we also trained two multiclass SVM models. The first model was trained in a one-vs-all (i.e., SCZ vs. all, MDD vs. all, HC vs. all) repeated nested cross-validation structure with 10 folds and 10 permutations at each level. Similarly, the second multiclass model was trained in a one-vs-one setup in the same cross-validation structure. The model training process was optimized for multiclass classification performance based on their error correcting output codes, which were calculated using the Hamming distance. In the one-vs-all setup, the classes were, expectedly, unbalanced, therefore a hyperplane weighting procedure was implemented to adjust for this label imbalance

## **Model Investigation**

Two main measures for the features used to determine the significance of the features that are included in the model are the overall mean of the cross-validation ratio and the sign-based consistency. The cross-validation ratio (CVR) illustrates the feature consistency uniformity of features over different cross-validation folds. It is calculated by summing the median weights from chosen inner folds across all outer folds and then dividing by the standard error of these selected inner weights. Sign-based consistency measures the significance of each input variable by assessing the number of times the sign (positive or negative) of a feature aligns consistently within a dataset, multiplied by the frequency with which the variable is non-zero. This metric yields a value between 0 and 1, where 1 signifies perfect alignment within the ensemble of data, and 0 suggests that the feature weights are evenly distributed between positive and negative or when the feature is entirely disregarded with a weight of zero. To evaluate the statistical significance of this importance score, a p-value is calculated. This involves setting up a hypothesis test with a null hypothesis stating the importance score is zero and an alternative hypothesis suggesting otherwise. A Z-score was calculated as the importance divided by the square root of the variance of the importance scores. A standard p-value was then calculated using a normal cumulative distribution function to choose the right-tailed significance. Finally, to address multiple comparisons, the p-values are corrected using the false discovery rate method. For more details, please see Koutsouleris et al, 2021, Supplementary Methods).

In the power spectrum density modality, the features that have sign-based consistency below the significance level (log(0.05)) were excluded, and then the median CVRs between every frequency interval (delta (1-3 Hz), theta (4-7 Hz), alpha (8-11 Hz), beta (12-24 Hz)), low gamma (25-39 Hz) and high gamma (40-70 Hz)) are calculated. The median CVRs that are above 2 and below -2 are regarded as significant. In connectivity modality, the features that have less than a significant level of sign-based consistency are regarded as non-informative, and with the resting significant feature overall mean CVRs, a graph network was built using the MATLAB “graph” function. The CVRs between two channels represented their edge and the channels were the graph’s nodes. The centrality in the graph network was calculated using Eigenvector Centrality. It evaluates a node's "centrality" by looking at both the number and quality of its connections with other key nodes in the network, offering a comprehensive view of its importance^4^.

## **Association between chronological age and power spectrum density**

To assess the effects of chronological age on EEG measures, we calculated Pearson correlation coefficients between the mean power spectrum density measurements at each frequency band and the chronological age of participants in central channels (Fz, Cz and Pz) for HC, MDD and SCZ individuals. The p-values are false discovery rate corrected for multiple comparisons^5^.

**Potential of EphysAGE as an additional predictor feature**

The predictive value of EphysAGE was evaluated by adding it to the three support vector machine classification models in two different way. First, we added EphysAGE as an additional data modality, i.e. a one-feature data modality, and trained an SVM model exclusively on EphysAGE for all three classification models. We then assessed the added value of EphysAGE by stacking the PSD, connectivity and EphysAGE model into a meta-learner. The general machine learning pipeline remained the same, as in the original MDD/SCZ classification models.

In a second approach, we added EphysAGE as a single feature and employed an early fusion strategy. Specifically, we concatenated the PSD, connectivity and EphysAGE modalities and preprocessed this one matrix before entering the machine learning analysis step. Therefore, one model was trained on all three data modalities combined. This approach was implemented for all three classification models (MDD/SCZ model, differential diagnostic model).

Lastly, the original binary model performances (BAC) from all three classification models (SCZ Model, MDD Model and Differential Diagnostic Model) at CV2-level testing folds were compared with the ones form the stacked and early fusion EphysAGE-added models and the one-vs-one multigroup classifier of the respective group with Quade Test to assess if there were any significant difference between the models.

**Different Conditions in EEG Recordings**

In EEG recordings, eyes open and eyes closed conditions cause electrophysiological and topographical changes ^6,7^. It is widely known that especially the alpha band frequency band power is suppressed during eyes-open condition with visual stimuli. Including only the eyes closed condition restricted our analysis with regards to electrophysiological changes and differences caused by the perceptual level abnormalities and disruptions in SMI. Further studies should explore the possibly notable differences between eyes open and eyes closed conditions.

# **Supplementary Results**

We did not observe any significant interaction between age and medication effects in our sample. Specifically, the rank transformed decision scores from the SVM models distinguishing medicated vs. unmedicated patients were not significantly different between older and younger SCZ (p=0.96) or older and younger MDD (p=0.54) patients. Moreover, we did not find any significant correlations between the CPZ or FLUOX dosage equivalents and EphysAGE the older and younger age dependent groups (RhoSCZ/Old= -0.10, pSCZ/Old=0.40; RhoSCZ/Young=0.01, pSCZ/Young=0.91; RhoMDD/Old=0.04, pMDD/Old=0.78; RhoMDD/Young=-0.01, pMDD/Young=0.28).

The one-vs-all multiclass model performed at chance level: SCZ vs. all (BAC=36.2.0%, SEN=38.8%, SPEC=33.6% , AUC[95%-CI]=0.33[0.29-0.37]), MDD vs. all (BAC=58.5%, SEN=53.3%, SPEC=61.2%, AUC[95%-CI]=0.61[0.57-0.65]), HC vs all (BAC=48.0%, SEN=50.2%, SPEC=45.7%, AUC=0.45[0.41-0.49]). The performances of this multiclass model was much lower than the performances of the binary classifiers, which might be due to the increased heterogeneity of the ‘all’ groups, which, depending on the setup, contained HC and MDD, HC and SCZ or MDD and SCZ. Upon further investigation of the confusion matrix and the misclassification web (Figure S1 and Figure S2) it emerged that the low classification performance was mainly driven by the misclassification of HC and SCZ as MDD individuals. Taken together with the cross-over application results, we regarded this as a further support for the gradual change between SCZ and MDD with increasing and overlapping chronological and pathological aging effects.

In contrast, the one-vs-one multiclass model performed at a similar level like the binary classifiers: SCZ vs. HC (BAC=72.9%, SEN=72.8%, SPEC=73.1%, AUC[95%-CI]=0.81[0.77-0.84]), MDD vs. HC (BAC=68.1%, SEN=70.8%, SPEC=65.3%, AUC[95%-CI]=0.73[0.68-0.77]), SCZ vs MDD (BAC=58.5%, SEN=60.8%, SPEC=56.2%, AUC=0.63[0.58-0.68]). Since the one-vs-one multi-class approach performs a pairwise classification between two distinct groups, it is rather similar to the binary models, which, in turn, explains the similar performances. Again, in the one-vs-one multiclass model, the SCZ and HC individuals were rather frequently misclassified as MDD individuals (One-vs-One Multiclass Model, Confusion Matrix: Figure S3 and Missclassification Web: Figure S4). Additionally, the difference between the two multiclass SVM Models was assessed through a Quade Test. The one-vs-one multiclass classification model performed significantly better than the multiclass model that was trained with a one-vs-all strategy (Qgrand-mean=0, F=4.02e+33, W=16.72, p-value<0.001).

The model investigation results for power spectrum density modality (Table S3) and the connectivity modality (Table S4) as well as the centrality values extracted from the connectivity modality (Table S5) are presented below.

In Figure S1, the topographical plots show CVR ratios of power spectrum density features in the MDD model. Overall increased theta and high gamma frequency powers were indicative of SCZ when discriminating them from MDD patients. Moreover, in Figure S2, the topographical representations of increased central delta activity and decreased peripheral high gamma activity predicting higher age suggesting an overall slowing in the neural oscillations with age.

We found a positive correlation between higher frequency band power values and chronological age in SCZ patients as well as a positive overall correlation in MDD patients in the Pz channel. (Table S6). These results might further support the need to use multivariate methods such as deep and shallow machine learning algorithms to disentangle the effects of aging which would enable us to reveal and target the neurophysiological correlates of severe mental illnesses.

The SVM models trained only on EphysAGE did not yield a performance above chance level. Specifically, the BACs for the differential diagnostic model as well as the SCZ and MDD models were 51.5%, 56.8% and 49.9%, respectively, with none of them being statistically significant. Therefore, they were not stacked with the other two feature modalities. The models that employed the early fusion approach yielded similar performances as the original models: SCZ vs. HC (BAC=73.9%, SEN=74.8%, SPEC=73.1% , AUC[95%-CI]=0.82[0.78-0.86]), MDD vs. HC (BAC=68.7%, SEN=69.2%, SPEC=68.2%, AUC[95%-CI]=0.73[0.69-0.77]), Differential Diagnosis (BAC=60.0%, SEN=58.0%, SPEC=62.1%, AUC=0.65[0.60-0.69]). Thus, the EphysAGE measure was a stable predictive feature only in Differential Diagnostic Model (SCZ vs. HC EphysAGE CVR= -1.64; MDD vs. HC EphysAGE CVR= -0.42, Differential Diagnosis EphysAGE CVR: -2.15).

Lastly, Quade test results revealed that the only significant performance difference among the SCZ Models (Q(SCZ)grand mean=3.57e-18, F=5.22, W=0.02, p-value=0.002) was between the SCZ EphysAGE-added Models with stacking (Qstacking=-0.20) and early-fusion (Qearly-fusion=0.08) strategies (df=3, p-value=0.03). However, this difference was not significant after FDR correction (p-value(FDR-corrected)=0.14). There were no significant difference among the MDD Models (Q(MDD)grand mean=-1.21e-17, F=0.81, W=0.004, p-value=0.49). Among the models that differentiate between the SCZ and MDD patients, there were significant differences (Q(DD)grand mean=-1.38e-17, F=31.44, W=0.12, p-value<0.001). The differential diagnostic EphysAGE-added model with stacking strategy (Qstacking=0.13) performed better than the one with early fusion strategy (Qearly-fusion=-0.19, p-value(FDR-corrected)=0.02) and the original binary classifier (Qbinary=-0.31, p-value(FDR-corrected)=0.002). The differential diagnostic classifier in the one-vs-one multigroup model (Qone-vs-one=0.37) performed significantly better than the original binary classifier (Qbinary=-0.31, p-value(FDR-corrected)<0.001). These findings suggest that EphysAGE may be most effective when used in conjunction with other features rather than as sole predictor.”

# **Supplementary Tables**

# **Table S1: Sociodemographic characteristics of the study sample**

Group compositions are provided based on their age, sex and medication intake information. FLUOX: Fluoxetine equivalent dose, CPZE: Chlorpromazine equivalent dose.

|  | **HC** | **SCZ** | **MDD** |
| --- | --- | --- | --- |
| **Sample Size (N)** | 245 | 250 | 240 |
| **16-25 years (%)** | 19 (7.78) | 46 (18.40) | 24 (10.00) |
| **26-35 years (%)** | 50 (20.41) | 77 (30.80) | 45 (18.75) |
| **36-45 years (%)** | 39 (15.92) | 79 (31.60) | 64 (26.67) |
| **46-55 years (%)** | 41 (16.73) | 32 (12.80) | 47 (19.58) |
| **56-65 years (%)** | 92 (37.55) | 14 (5.60) | 44 (18.33) |
| **Male (%)** | 124 (51.12) | 157 (62.80) | 95 (39.39) |
| **Female (%)** | 121 (48.88) | 93 (37.20) | 145 (60.61) |
| **Age (years, ±SD)** | 47.02 ±14.46 | 36.45 ±11.40 | 44.29 ±14.44 |
| **Medicated (%)** | N/A | 126 (50.40) | 84 (35.00) |
| **Non-Medicated (%)** | N/A | 90 (36.00) | 146 (60.83) |
| **FLUOX (mg, ±SD)** | N/A | N/A | 39.31±23.23 |
| **CPZE (mg, ±SD)** | N/A | 354.3 ±212.44 | N/A |

**Table S2: Source Studies of the Participants**

| **Group** | **Rebox** | **MRP300** | **Katamnese Schizophrenie** | **Katamnese Depression** | **Remergil** | **TMS** | **Genetik** | **Akut** | **Basis-Schizophrenie** | **DynSpect** | **Routine** |
| --- | --- | --- | --- | --- | --- | --- | --- | --- | --- | --- | --- |
| **SCZ** | 0 | 31 | 19 | 0 | 0 | 0 | 128 | 37 | 15 | 17 | 2 |
| **MDD** | 130 | 0 | 0 | 58 | 9 | 10 | 0 | 0 | 0 | 0 | 33 |
| **HC** | 0 | 0 | 0 | 0 | 0 | 0 | 245 | 0 | 0 | 0 | 0 |

**Table S3: Overall mean CVR for power spectrum density modality in all significant models**

The mean CVR values are displayed for every channel in their respective frequency intervals. The significant values (ABS(CVR)>2) are indicated in bold.

| **Frequency Interval (Hz)** | **Fp1** | **Fp2** | **F3** | **F4** | **C3** | **C4** | **P3** | **P4** | **O1** | **O2** | | **F7** | **F8** | **T3** | | **T4** | **T5** | **T6** | **Fz** | **Cz** | **Pz** | |  |
| --- | --- | --- | --- | --- | --- | --- | --- | --- | --- | --- | --- | --- | --- | --- | --- | --- | --- | --- | --- | --- | --- | --- | --- |
| **SCZ Model** | | | | | | | | | | |  |  |  |  |  |  |  |  |  |  |  |  |  |
| Delta  (1-3) | 0.90 | 0.04 | -0.90 | 0.90 | 0.04 | 0.04 | 0.04 | 1.84 | 0.04 | -0.90 | | 0.04 | 0.90 | 0.04 | | 0.04 | NaN | 1.84 | 0.04 | -0.90 | 0.04 | |  |
| Theta  (4-7) | 1.56 | 0.50 | 1.03 | 1.03 | 0.50 | 1.91 | 1.03 | 0.50 | 1.03 | 0.50 | | 1.56 | 0.50 | 1.03 | | 1.03 | -1.46 | -0.58 | -0.93 | 0.50 | 1.03 | |  |
| Alpha  (8-11) | -1.50 | -1.50 | **-2.14** | **-2.14** | **-4.78** | **-3.46** | -0.85 | **-2.14** | **-2.14** | -1.50 | | -1.50 | -1.50 | -1.50 | | -1.50 | -0.85 | -1.50 | **-2.14** | -1.50 | -1.50 | |  |
| Beta  (12-19) | -0.70 | 0.12 | -0.53 | 0.36 | 0.52 | -0.70 | 0.12 | -0.11 | 0.52 | 0.36 | | -0.11 | 0.20 | -0.40 | | 0.00 | -0.23 | 0.36 | -0.36 | 0.43 | 0.12 | |  |
| Low Gamma  (20-49) | 1.14 | 0.27 | 0.04 | 0.26 | 0.71 | 0.27 | 0.15 | 0.27 | 0.27 | 0.69 | | 0.26 | 0.27 | 0.71 | | 0.27 | -0.09 | -0.09 | 0.27 | 0.15 | 0.15 | |  |
| High Gamma  (50-70) | 1.25 | 0.75 | 0.70 | 1.21 | 0.93 | 0.75 | 1.14 | 0.84 | 0.83 | 0.56 | | 1.32 | 1.65 | 0.66 | | 1.02 | 0.83 | 1.14 | 1.25 | 0.83 | 0.66 | |  |
| **MDD Model** | | | | | | | | | | | | | | |  |  |  |  |  |  |  |  |  |
| Delta  (1-3) | **2.61** | **2.61** | **2.61** | 1.15 | 1.15 | **2.91** | 1.15 | **2.61** | **2.61** | **2.61** | | 1.15 | **2.61** | **2.91** | | 0.85 | **3.21** | **3.21** | **2.61** | **2.61** | **2.61** | |  |
| Theta  (4-7) | -0.58 | -0.58 | -1.19 | -0.58 | -0.58 | 1.40 | -0.44 | -1.05 | 1.40 | -0.58 | | **-2.56** | -1.19 | -0.58 | | -0.58 | -0.58 | -0.58 | **-2.56** | **-2.56** | -0.58 | |  |
| Alpha  (8-11) | -0.65 | -0.65 | -1.96 | -1.96 | -1.96 | **-2.07** | -0.65 | -0.65 | 0.66 | 0.66 | | 1.66 | -1.96 | -0.65 | | -0.65 | -1.96 | -1.96 | -0.65 | 0.66 | -0.27 | |  |
| Beta  (12-19) | -1.47 | -0.38 | -0.41 | -0.76 | -0.60 | -0.41 | -0.60 | -0.38 | -0.41 | -0.43 | | -0.38 | -0.60 | -0.41 | | -0.60 | -0.43 | 0.06 | -0.38 | -0.41 | 0.08 | |  |
| Low Gamma (20-49) | -0.85 | 0.46 | 0.46 | -0.11 | 0.43 | 0.46 | -0.11 | 0.24 | -0.46 | -0.46 | | 0.43 | 0.39 | 0.39 | | 0.35 | 0.24 | -0.52 | 0.39 | -0.03 | 0.24 | |  |
| High Gamma (50-70) | -1.16 | 0.54 | -0.08 | -0.68 | -1.55 | -0.01 | -0.62 | -0.76 | -0.08 | 0.30 | | -0.01 | -0.82 | 0.54 | | 0.07 | 0.07 | 0.07 | -0.08 | 0.07 | -0.62 | |  |
| **Differential Diagnostic Model** | | | | | | | | | | | | | | | | | | | | | | | |
| Delta  (1-3) | -0.39 | -0.25 | -0.25 | -0.25 | -1.64 | 1.00 | -0.25 | 1.00 | -0.25 | -0.39 | | -0.25 | 1.00 | -0.25 | | -0.25 | -0.25 | 1.00 | 1.00 | -1.64 | -0.39 | |  |
| Theta  (4-7) | 1.71 | 1.71 | **2.06** | 1.71 | 1.37 | -0.58 | 1.71 | 1.37 | 1.71 | **2.36** | | 1.71 | **2.06** | 1.37 | | **2.06** | 1.37 | **2.06** | 1.71 | 1.71 | 1.71 | |  |
| Alpha  (8-11) | 1.41 | 0.43 | 1.41 | -0.54 | 0.43 | 0.79 | 1.77 | 1.41 | -0.54 | 0.43 | | -0.54 | -0.54 | 0.43 | | 1.41 | -0.54 | -0.54 | 0.43 | 0.43 | 0.43 | |  |
| Beta  (12-19) | -0.06 | 0.00 | -0.59 | -0.61 | 0.00 | -0.59 | -0.62 | -0.59 | 0.22 | -0.59 | | -0.59 | 0.22 | 0.00 | | 0.22 | -0.29 | -0.61 | -0.61 | -0.59 | -0.59 | |  |
| Low Gamma (20-49) | -0.94 | 0.53 | 0.32 | -0.92 | 0.86 | -0.10 | 0.32 | -0.28 | -0.03 | -0.28 | | -0.48 | 0.14 | -0.52 | | -0.03 | -0.10 | 0.14 | -0.28 | 0.42 | -0.72 | |  |
| High Gamma (50-70) | 1.94 | 1.94 | 1.84 | 1.18 | 1.18 | 1.35 | 1.68 | 1.68 | 1.35 | 1.52 | | 1.84 | 1.52 | 1.81 | | **2.44** | 1.52 | **2.04** | 1.52 | **2.14** | 1.84 | |  |
| **EphysAGE Model** | | | | | | | | | | | | | | | | | | | | | |  |  |
| Delta  (1-3) | **2.32** | **2.32** | **2.32** | **3.98** | **2.32** | **2.32** | **3.98** | **2.32** | **2.32** | 1.54 | | **2.32** | **2.32** | **2.32** | | 1.54 | **2.32** | **2.37** | **2.32** | **3.98** | 1.54 | |  |
| Theta  (4-7) | 1.28 | 0.44 | 1.28 | 0.44 | 0.44 | 0.44 | 1.28 | 0.44 | -0.76 | 1.28 | | 0.44 | -0.41 | 0.44 | | 0.44 | 1.28 | 1.28 | -0.76 | 0.44 | 1.28 | |  |
| Alpha  (8-11) | 1.65 | 0.73 | 0.73 | -0.18 | 0.73 | -0.18 | 0.73 | 1.65 | 1.65 | 1.65 | | -1.24 | -0.18 | -0.18 | | 0.79 | 0.73 | 1.65 | 0.79 | 0.73 | 0.73 | |  |
| Beta  (12-19) | 1.62 | 0.70 | 0.95 | 0.77 | 0.57 | 0.77 | 1.14 | 0.77 | 0.70 | 0.77 | | 0.47 | 1.58 | 0.77 | | 0.70 | 0.62 | 0.95 | 0.95 | 1.14 | 1.14 | |  |
| Low Gamma (20-49) | -0.84 | -0.30 | 0.10 | 0.21 | 0.16 | 0.18 | 0.16 | 0.01 | 0.17 | 0.16 | | -0.05 | 0.17 | 0.10 | | 0.18 | 0.17 | 0.17 | -0.05 | 0.18 | 0.10 | |  |
| High Gamma (50-70) | **-2.04** | -0.33 | -0.80 | **-2.02** | -1.41 | -0.67 | -0.80 | -1.27 | -1.88 | -1.88 | | -1.27 | **-2.04** | -1.74 | | -0.33 | **-2.02** | -1.14 | -0.80 | -1.14 | -0.12 | |  |

**Table S4: Overall mean CVR for connectivity modality in all significant models**

The CVR values for connectivity modality are provided and the significant values (ABS(CVR)>2) are displayed in bold.

| **SCZ Model** | | | | | | | | | | | | | | | | | | | |
| --- | --- | --- | --- | --- | --- | --- | --- | --- | --- | --- | --- | --- | --- | --- | --- | --- | --- | --- | --- |
|  | **Fp1** | **Fp2** | **F3** | **F4** | **C3** | **C4** | **P3** | **P4** | **O1** | **O2** | **F7** | **F8** | **T3** | **T4** | **T5** | **T6** | **Fz** | **Cz** | **Pz** |
| **Fp1** |  |  |  |  |  |  |  |  |  |  |  |  |  |  |  |  |  |  |  |
| **Fp2** | **-4.06** |  |  |  |  |  |  |  |  |  |  |  |  |  |  |  |  |  |  |
| **F3** | -0.70 | 1.40 |  |  |  |  |  |  |  |  |  |  |  |  |  |  |  |  |  |
| **F4** | 1.22 | **-2.12** | 0.99 |  |  |  |  |  |  |  |  |  |  |  |  |  |  |  |  |
| **C3** | 1.44 | -- | -1.57 | 0.78 |  |  |  |  |  |  |  |  |  |  |  |  |  |  |  |
| **C4** | **-2.64** | **-3.93** | **-2.46** | **3.32** | -1.84 |  |  |  |  |  |  |  |  |  |  |  |  |  |  |
| **P3** | **2.37** | 1.02 | **3.59** | **3.27** | -- | 1.87 |  |  |  |  |  |  |  |  |  |  |  |  |  |
| **P4** | **-2.77** | 1.30 | -1.61 | -- | **-2.02** | -1.48 | 1.81 |  |  |  |  |  |  |  |  |  |  |  |  |
| **O1** | -- | -- | 1.80 | 1.19 | 0.91 | -- | -1.50 | -1.66 |  |  |  |  |  |  |  |  |  |  |  |
| **O2** | -1.53 | -- | -1.99 | -- | -1.05 | -- | -- | **-2.41** | -- |  |  |  |  |  |  |  |  |  |  |
| **F7** | **-3.17** | -- | **-2.08** | -0.87 | **2.32** | **-3.50** | -1.60 | -1.26 | -- | -0.69 |  |  |  |  |  |  |  |  |  |
| **F8** | -- | **2.34** | **-2.33** | **2.12** | **2.57** | -- | 0.62 | -- | **3.35** | **3.02** | **-3.35** |  |  |  |  |  |  |  |  |
| **T3** | **-2.96** | -- | -- | -- | -- | **5.71** | -1.70 | -0.99 | -0.74 | **-4.12** | **5.31** | -- |  |  |  |  |  |  |  |
| **T4** | **2.99** | 1.92 | 0.86 | -- | **2.01** | **2.69** | -- | 2.00 | 0.84 | 1.16 | 0.82 | -- | -1.45 |  |  |  |  |  |  |
| **T5** | 1.30 | -- | 0.96 | -- | **-2.47** | 1.79 | **2.77** | -0.88 | **3.98** | -1.72 | -1.95 | -- | 1.29 | -0.96 |  |  |  |  |  |
| **T6** | 1.55 | 0.88 | 0.97 | -1.52 | **-3.03** | **-2.09** | **-2.40** | **3.37** | -- | 1.89 | **5.93** | -0.98 | 1.59 | 0.85 | -- |  |  |  |  |
| **Fz** | -- | **-2.75** | **2.71** | -1.89 | -- | -- | -- | -1.33 | 1.56 | -- | 1.80 | **2.51** | -1.19 | 1.24 | **2.33** | **3.39** |  |  |  |
| **Cz** | -1.10 | -0.93 | -1.79 | **-3.91** | **2.28** | **-3.06** | -- | 0.35 | -1.75 | -0.96 | **-2.48** | -1.26 | **-2.43** | -- | **-4.35** | -- | -0.97 |  |  |
| **Pz** | **3.23** | **3.14** | -0.76 | -- | -1.09 | **-2.21** | -0.94 | 5.21 | **-4.19** | -1.28 | **-2.19** | -1.29 | -1.57 | -0.91 | -1.61 | -- | -- | -1.02 |  |
| **MDD Model** | | | | | | | | | | | | | | | | | | | |
|  | **Fp1** | **Fp2** | **F3** | **F4** | **C3** | **C4** | **P3** | **P4** | **O1** | **O2** | **F7** | **F8** | **T3** | **T4** | **T5** | **T6** | **Fz** | **Cz** | **Pz** |
| **Fp1** |  |  |  |  |  |  |  |  |  |  |  |  |  |  |  |  |  |  |  |
| **Fp2** | **-5.51** |  |  |  |  |  |  |  |  |  |  |  |  |  |  |  |  |  |  |
| **F3** | -- | **3.51** |  |  |  |  |  |  |  |  |  |  |  |  |  |  |  |  |  |
| **F4** | 0.86 | **-2.65** | **2.82** |  |  |  |  |  |  |  |  |  |  |  |  |  |  |  |  |
| **C3** | 1.40 | 1.25 | **-2.66** | **2.70** |  |  |  |  |  |  |  |  |  |  |  |  |  |  |  |
| **C4** | **-2.71** | -0.88 | **-5.96** | **2.04** | -- |  |  |  |  |  |  |  |  |  |  |  |  |  |  |
| **P3** | **4.26** | **4.90** | **-2.21** | -- | **-3.37** | -- |  |  |  |  |  |  |  |  |  |  |  |  |  |
| **P4** | **-2.07** | **3.09** | -1.10 | 1.37 | -1.06 | **-3.35** | **2.69** |  |  |  |  |  |  |  |  |  |  |  |  |
| **O1** | **-2.05** | **-3.52** | -0.83 | **2.21** | -- | -1.74 | **-4.68** | -- |  |  |  |  |  |  |  |  |  |  |  |
| **O2** | **-6.35** | **-3.17** | -1.81 | **3.58** | 0.72 | **-2.09** | **4.55** | -1.75 | **2.60** |  |  |  |  |  |  |  |  |  |  |
| **F7** | **-8.93** | -- | **-2.14** | -- | -1.89 | **-3.69** | -0.77 | -- | -- | **2.37** |  |  |  |  |  |  |  |  |  |
| **F8** | -1.07 | 1.82 | -- | -- | -0.93 | -1.19 | -1.82 | 1.36 | **3.62** | **3.20** | -- |  |  |  |  |  |  |  |  |
| **T3** | **-4.39** | **-2.08** | **2.49** | -- | 0.77 | **7.92** | -- | **4.32** | **-4.69** | -- | **2.03** | -- |  |  |  |  |  |  |  |
| **T4** | 1.68 | 1.85 | 1.38 | **-2.69** | 1.96 | **2.60** | -- | **2.07** | 1.13 | 1.70 | **-3.79** | -- | **-4.40** |  |  |  |  |  |  |
| **T5** | **4.81** | -- | 1.42 | 1.85 | **-4.75** | 1.02 | **3.60** | -1.81 | **5.89** | -1.01 | -1.91 | **3.12** | 1.77 | -0.92 |  |  |  |  |  |
| **T6** | -- | -- | **2.71** | **3.36** | **-4.27** | **-2.49** | **-2.22** | **2.71** | 1.23 | -- | **8.68** | **-2.16** | **3.85** | **4.02** | -1.16 |  |  |  |  |
| **Fz** | **-2.02** | **-3.54** | -- | -- | **2.52** | **-2.03** | -1.50 | **2.04** | **-3.08** | -1.60 | **3.44** | **5.40** | -1.50 | 1.15 | **4.34** | **4.66** |  |  |  |
| **Cz** | -- | -- | -1.21 | -1.95 | **5.94** | -- | -1.80 | **2.11** | **-3.53** | -0.89 | **-5.68** | **-3.50** | **-3.27** | -- | **-3.25** | 1.64 | **-3.64** |  |  |
| **Pz** | -- | **4.80** | **-2.69** | -- | **-5.47** | **-5.59** | **-2.53** | **2.09** | **-5.87** | 1.17 | **-4.38** | 1.61 | -1.42 | -- | **-2.61** | 0.81 | **-2.13** | **-2.51** |  |
| **Differential Diagnostic Model (SCZ vs. MDD)** | | | | | | | | | | | | | | | | | | | |
|  | **Fp1** | **Fp2** | **F3** | **F4** | **C3** | **C4** | **P3** | **P4** | **O1** | **O2** | **F7** | **F8** | **T3** | **T4** | **T5** | **T6** | **Fz** | **Cz** | **Pz** |
| **Fp1** |  |  |  |  |  |  |  |  |  |  |  |  |  |  |  |  |  |  |  |
| **Fp2** | **2.21** |  |  |  |  |  |  |  |  |  |  |  |  |  |  |  |  |  |  |
| **F3** | -- | **2.25** |  |  |  |  |  |  |  |  |  |  |  |  |  |  |  |  |  |
| **F4** | -- | **2.90** | -1.02 |  |  |  |  |  |  |  |  |  |  |  |  |  |  |  |  |
| **C3** | **2.20** | -1.12 | **2.01** | **-2.67** |  |  |  |  |  |  |  |  |  |  |  |  |  |  |  |
| **C4** | **2.08** | -1.31 | 1.41 | **4.13** | 1.74 |  |  |  |  |  |  |  |  |  |  |  |  |  |  |
| **P3** | **2.41** | **2.01** | **3.38** | **4.77** | 1.12 | **2.93** |  |  |  |  |  |  |  |  |  |  |  |  |  |
| **P4** | -- | 1.44 | -0.96 | 0.85 | **-4.20** | **2.05** | **-2.62** |  |  |  |  |  |  |  |  |  |  |  |  |
| **O1** | **5.62** | **4.95** | **3.62** | -- | **2.17** | **2.19** | **2.93** | **-3.10** |  |  |  |  |  |  |  |  |  |  |  |
| **O2** | **2.72** | **2.37** | 1.60 | **-3.48** | 1.25 | -- | 1.69 | -- | 1.57 |  |  |  |  |  |  |  |  |  |  |
| **F7** | **4.17** | **2.19** | -- | 1.73 | **3.75** | **3.13** | **-3.88** | 1.87 | -0.58 | 1.54 |  |  |  |  |  |  |  |  |  |
| **F8** | -- | **2.73** | **-2.01** | **3.79** | **3.15** | **2.52** | **4.58** | **2.28** | -- | **2.28** | **-3.92** |  |  |  |  |  |  |  |  |
| **T3** | **2.40** | -0.44 | **2.36** | 1.98 | **3.87** | **2.04** | **2.41** | -1.36 | **2.61** | **-2.51** | **3.99** | -- |  |  |  |  |  |  |  |
| **T4** | **2.38** | -- | 1.87 | **4.16** | **2.77** | **4.02** | **2.65** | -- | **2.16** | -0.83 | **2.86** | 0.95 | **2.86** |  |  |  |  |  |  |
| **T5** | **-4.07** | 1.90 | **2.92** | **2.00** | 0.65 | **2.03** | **2.29** | 1.71 | 0.75 | 1.72 | **-2.12** | 1.57 | 0.98 | **2.23** |  |  |  |  |  |
| **T6** | -1.38 | 1.95 | 1.19 | 1.10 | **3.20** | **2.18** | -1.90 | **2.43** | -- | **5.23** | -- | **2.02** | -1.28 | -- | -1.07 |  |  |  |  |
| **Fz** | **2.52** | 1.64 | **3.73** | -1.42 | 1.14 | 1.33 | **2.97** | -1.42 | **5.19** | **3.92** | 1.46 | **-3.77** | 1.65 | **-3.67** | **2.16** | **-3.66** |  |  |  |
| **Cz** | -- | **-3.58** | 1.72 | **-2.00** | 1.65 | **-4.12** | 1.74 | **-6.53** | **2.18** | -- | **2.68** | -1.35 | **2.14** | 0.82 | **2.03** | -0.90 | **2.65** |  |  |
| **Pz** | **6.58** | 1.10 | 1.43 | 1.65 | -- | 1.13 | -- | **3.87** | -0.97 | 0.52 | 1.27 | 0.87 | -0.84 | 1.33 | **-2.05** | **2.40** | -- | -- |  |
| **EphysAGE Model** | | | | | | | | | | | | | | | | | | | |
|  | **Fp1** | **Fp2** | **F3** | **F4** | **C3** | **C4** | **P3** | **P4** | **O1** | **O2** | **F7** | **F8** | **T3** | **T4** | **T5** | **T6** | **Fz** | **Cz** | **Pz** |
| **Fp1** |  |  |  |  |  |  |  |  |  |  |  |  |  |  |  |  |  |  |  |
| **Fp2** | **-5.07** |  |  |  |  |  |  |  |  |  |  |  |  |  |  |  |  |  |  |
| **F3** | **4.45** | **2.11** |  |  |  |  |  |  |  |  |  |  |  |  |  |  |  |  |  |
| **F4** | **2.44** | 0.60 | **-3.22** |  |  |  |  |  |  |  |  |  |  |  |  |  |  |  |  |
| **C3** | **2.10** | **-2.29** | -- | -0.72 |  |  |  |  |  |  |  |  |  |  |  |  |  |  |  |
| **C4** | **-2.43** | -- | **3.53** | 1.24 | -- |  |  |  |  |  |  |  |  |  |  |  |  |  |  |
| **P3** | -0.62 | **-3.84** | 0.83 | -1.35 | **4.34** | 0.90 |  |  |  |  |  |  |  |  |  |  |  |  |  |
| **P4** | -1.25 | 0.71 | **2.39** | 1.79 | 1.77 | 1.76 | 0.97 |  |  |  |  |  |  |  |  |  |  |  |  |
| **O1** | 1.22 | 1.22 | **-2.56** | -1.16 | 1.84 | **-3.34** | **-5.56** | -1.81 |  |  |  |  |  |  |  |  |  |  |  |
| **O2** | **2.85** | 0.84 | -1.37 | -- | **-5.69** | **2.67** | -- | **-5.46** | -- |  |  |  |  |  |  |  |  |  |  |
| **F7** | **-2.41** | **-3.78** | -- | **2.88** | **2.36** | **-4.04** | **2.48** | **-5.93** | -1.68 | -1.38 |  |  |  |  |  |  |  |  |  |
| **F8** | -1.91 | -- | 1.09 | **2.34** | **2.67** | **-2.02** | 1.86 | **2.00** | 1.37 | **3.59** | **-4.23** |  |  |  |  |  |  |  |  |
| **T3** | -0.85 | -- | -1.75 | -1.17 | **-9.78** | **3.48** | **-2.01** | **-5.80** | **2.34** | **-5.59** | **7.00** | **4.78** |  |  |  |  |  |  |  |
| **T4** | **3.34** | **3.10** | -- | -0.69 | -- | **-6.12** | **-3.54** | 1.50 | 1.76 | 1.53 | -- | -- | -1.21 |  |  |  |  |  |  |
| **T5** | 1.01 | -- | -1.52 | **-4.96** | -1.74 | **-4.13** | -1.89 | -1.05 | **2.60** | -1.19 | 1.18 | **10.70** | 1.21 | -1.34 |  |  |  |  |  |
| **T6** | 1.55 | -0.85 | -1.82 | -- | **-3.26** | 0.90 | **-3.15** | -- | -1.38 | 1.79 | -1.40 | 0.67 | **-3.06** | **5.10** | **-3.49** |  |  |  |  |
| **Fz** | **3.40** | 1.98 | -- | **-3.25** | -- | 1.06 | **3.83** | 1.74 | -- | 1.40 | **3.83** | **5.94** | -- | 1.50 | -- | 0.69 |  |  |  |
| **Cz** | **2.43** | -- | -- | **-2.61** | -0.74 | -0.73 | 1.08 | **2.12** | **2.18** | -- | **-3.80** | 0.77 | 1.46 | 0.88 | -- | -1.05 | 1.32 |  |  |
| **Pz** | 1.27 | **3.03** | -- | 1.51 | **2.69** | 0.72 | 0.98 | -0.83 | -1.23 | -- | **-5.15** | 0.86 | -- | 1.38 | 0.82 | -0.63 | **3.19** | -1.58 |  |

**Table S5: Centrality Values in Connectivity Modalities**

|  | **SCZ Model** | **MDD Model** | **SCZ vs. MDD Model** | **EphysAGE Model** |
| --- | --- | --- | --- | --- |
| Fp1 | 0.07 | 0.06 | 0.07 | 0.07 |
| Fp2 | 0.06 | 0.06 | 0.05 | 0.05 |
| F3 | 0.05 | 0.05 | 0.05 | 0.04 |
| F4 | 0.05 | 0.04 | 0.04 | 0.05 |
| C3 | 0.06 | 0.04 | 0.06 | 0.07 |
| C4 | 0.09 | 0.06 | 0.07 | 0.07 |
| P3 | 0.04 | 0.06 | 0.07 | 0.06 |
| P4 | 0.05 | 0.06 | 0.04 | 0.05 |
| O1 | 0.02 | 0.06 | 0.06 | 0.04 |
| O2 | 0.02 | 0.04 | 0.04 | 0.05 |
| F7 | 0.09 | 0.06 | 0.06 | 0.09 |
| F8 | 0.06 | 0.03 | 0.06 | 0.07 |
| T3 | 0.05 | 0.06 | 0.06 | 0.07 |
| T4 | 0.04 | 0.03 | 0.06 | 0.04 |
| T5 | 0.03 | 0.04 | 0.05 | 0.03 |
| T6 | 0.06 | 0.06 | 0.03 | 0.03 |
| Fz | 0.04 | 0.07 | 0.06 | 0.05 |
| Cz | 0.06 | 0.05 | 0.05 | 0.04 |
| Pz | 0.06 | 0.06 | 0.02 | 0.03 |

**Table S6: Correlations between Power Spectrum Density and Chronological Age**

P values were adjusted for multiple comparisons using the False-Discovery Rate (PFDR).

|  | **SCZ** | | **MDD** | | **HC** | |
| --- | --- | --- | --- | --- | --- | --- |
|  | **Rho** | **p-value** | **Rho** | **p-value** | **Rho** | **p-value** |
| **Fz Channel** | | | | | | |
| Delta | 0.06 | 0.45 | 0.11 | 0.09 | -0.15 | 0.09 |
| Theta | 0.17 | **0.04** | 0.13 | 0.06 | 0.04 | 0.54 |
| Alpha | 0.04 | 0.54 | 0.10 | 0.12 | -0.12 | 0.21 |
| Beta | 0.15 | **0.04** | 0.16 | 0.05 | -0.08 | 0.23 |
| Low Gamma | 0.14 | **0.04** | 0.14 | 0.05 | -0.10 | 0.22 |
| High Gamma | 0.15 | **0.04** | 0.15 | 0.05 | -0.09 | 0.22 |
| **Cz Channel** | | | | | | |
| Delta | 0.08 | 0.25 | 0.11 | 0.10 | -0.07 | 0.29 |
| Theta | 0.07 | 0.27 | 0.11 | 0.10 | -0.22 | **0.00** |
| Alpha | 0.13 | 0.06 | 0.08 | 0.23 | -0.08 | 0.24 |
| Beta | 0.15 | **0.04** | 0.16 | 0.05 | -0.11 | 0.15 |
| Low Gamma | 0.15 | **0.04** | 0.14 | 0.07 | -0.10 | 0.15 |
| High Gamma | 0.15 | **0.04** | 0.15 | 0.05 | -0.11 | 0.15 |
| **Pz Channel** | | | | | | |
| Delta | 0.06 | 0.33 | 0.14 | **0.04** | -0.11 | 0.13 |
| Theta | 0.12 | 0.09 | 0.06 | 0.32 | -0.07 | 0.30 |
| Alpha | 0.09 | 0.18 | 0.19 | **0.02** | -0.10 | 0.14 |
| Beta | 0.14 | 0.07 | 0.15 | **0.03** | -0.11 | 0.13 |
| Low Gamma | 0.14 | 0.07 | 0.15 | **0.03** | -0.11 | 0.13 |
| High Gamma | 0.13 | 0.07 | 0.16 | **0.03** | -0.13 | 0.13 |


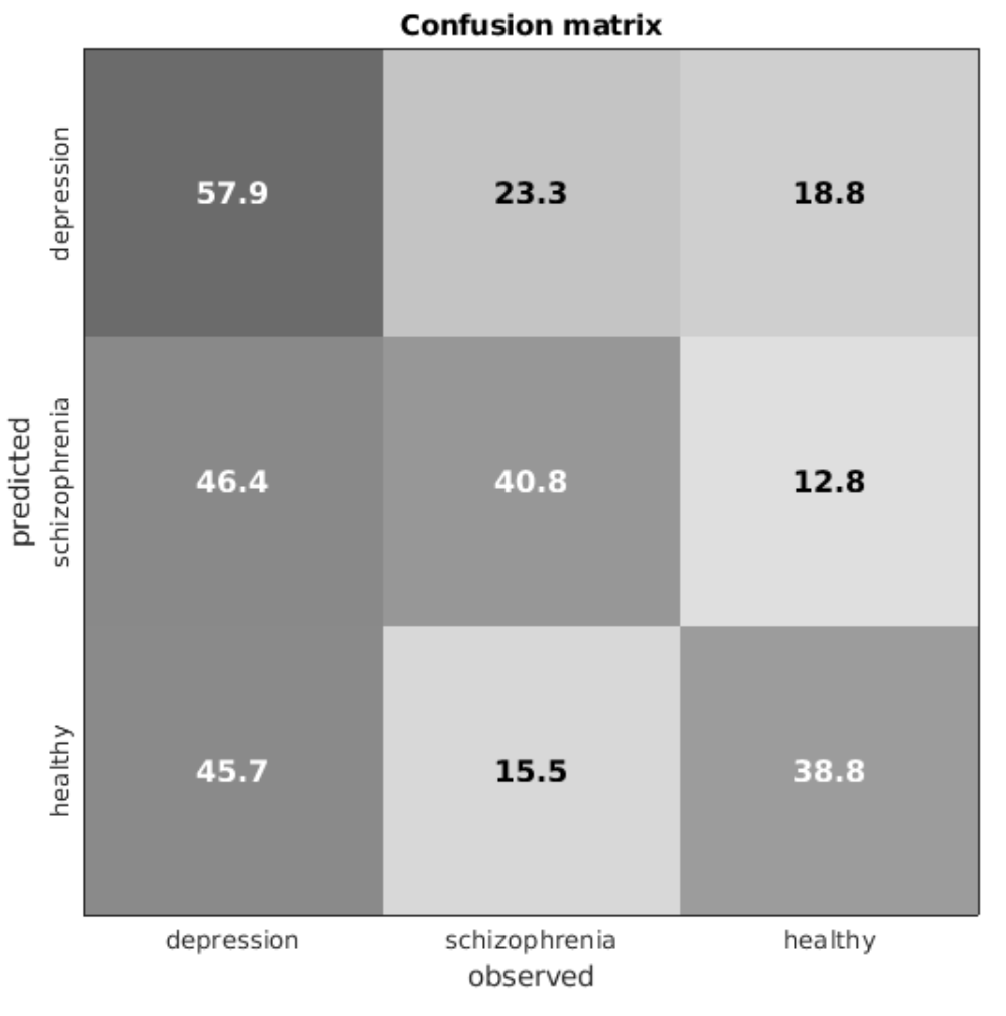


**Figure S1: One vs. All Multiclass Model Confusion Matrix**

Each row of the confusion matrix shows the percentage of the composition of the predicted group regarding their observed group assignment.


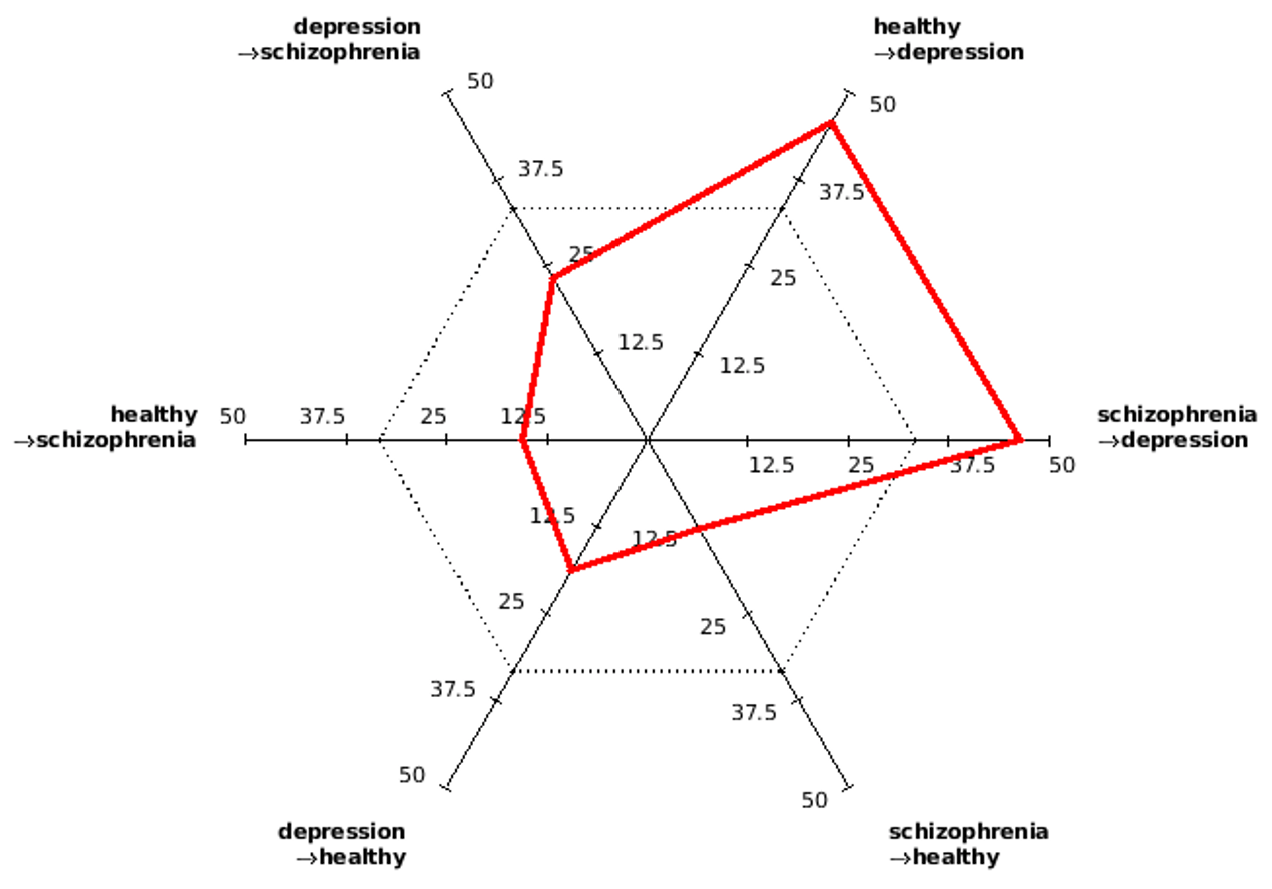


**Figure S2: One vs. All Multiclass Model Misclassification Web**

The misclassification web shows the percentage of cases from each observed group incorrectly classified as another specific group (e.g., the percentage of healthy cases misclassified as schizophrenia by the multiclass model).

**
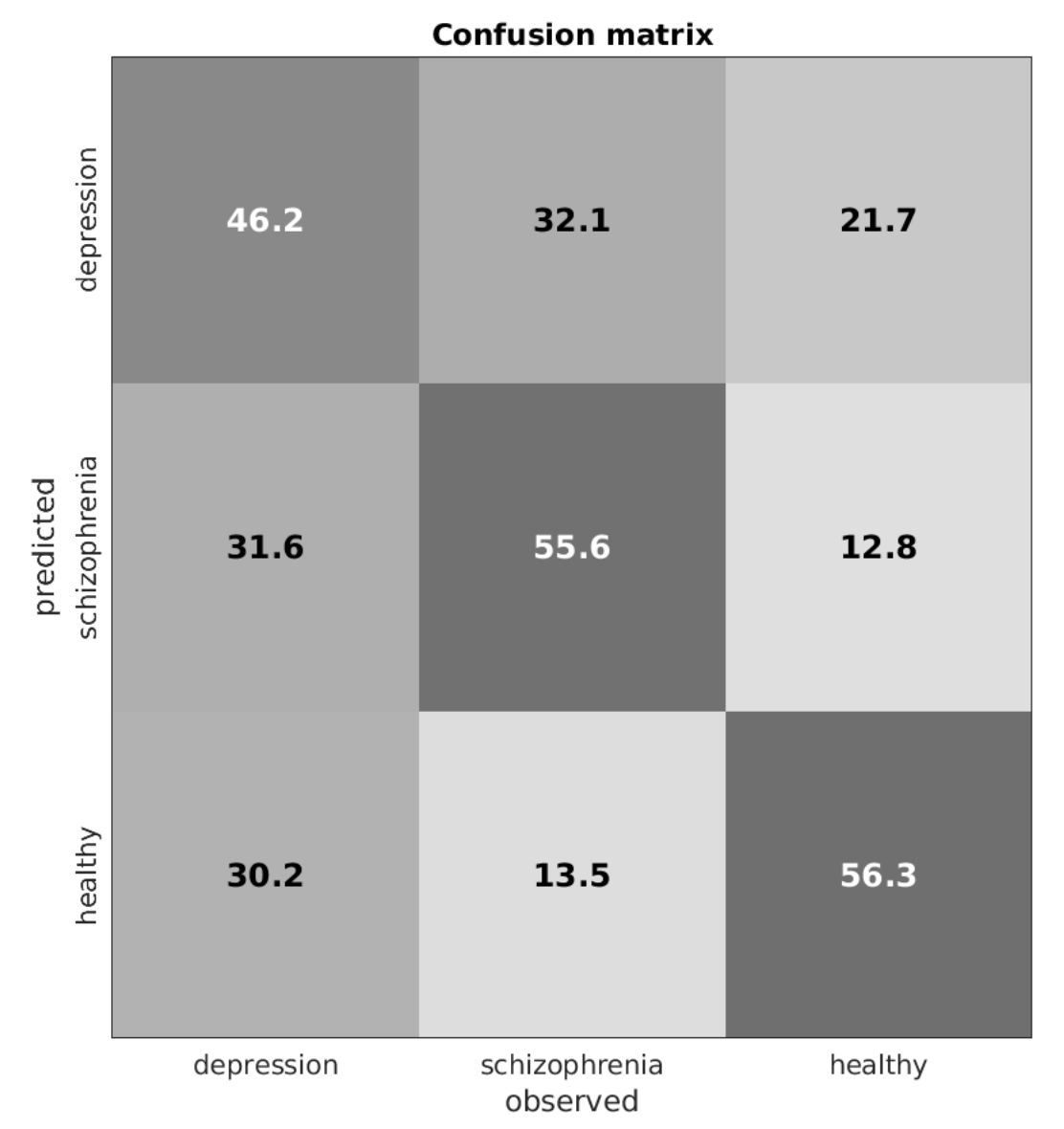
**

**Figure S3: One vs. One Multiclass Model Confusion Matrix**

Each row of the confusion matrix shows the percentage of the composition of the predicted group regarding their observed group assignment.

**
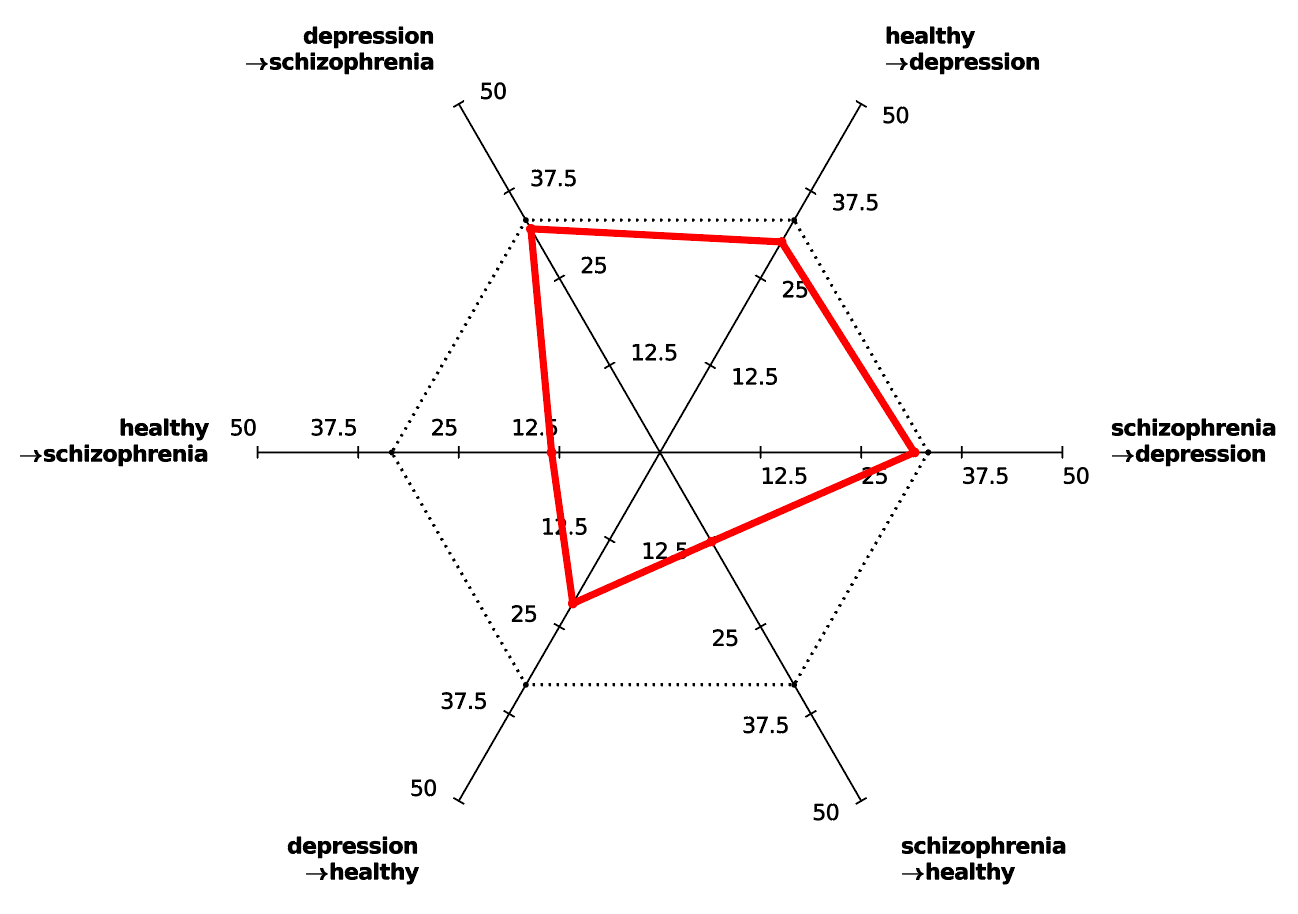
**

**Figure S4: One vs. One Multiclass Model Misclassification Web**

The misclassification web shows the percentage of cases from each observed group incorrectly classified as another specific group (e.g., the percentage of healthy cases misclassified as schizophrenia by the multiclass model).

**
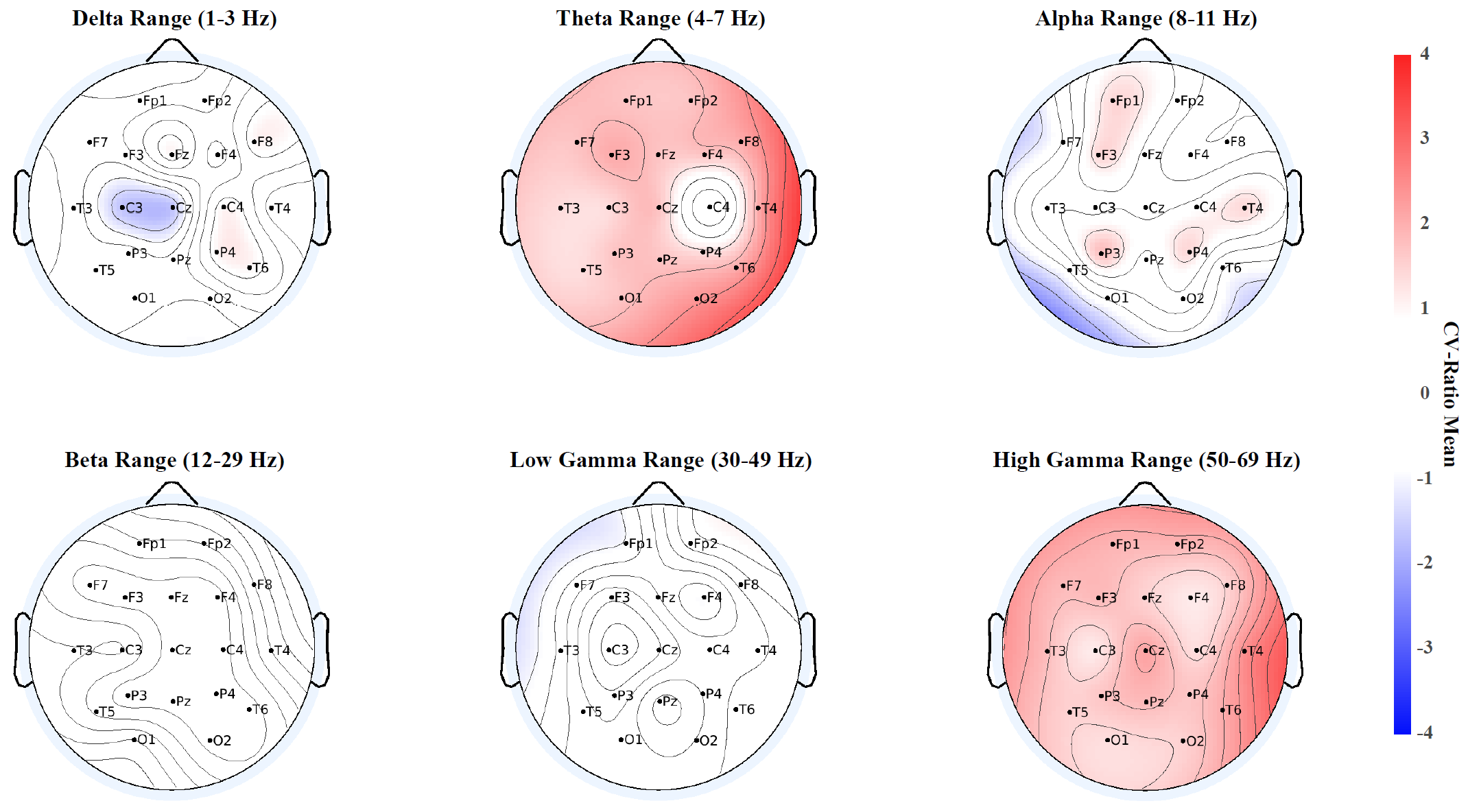
**

**Figure S5: Topographical Plots of Overall Mean of Cross-Validation Ratio in Classification Models - Differential Diagnostic Model**

**
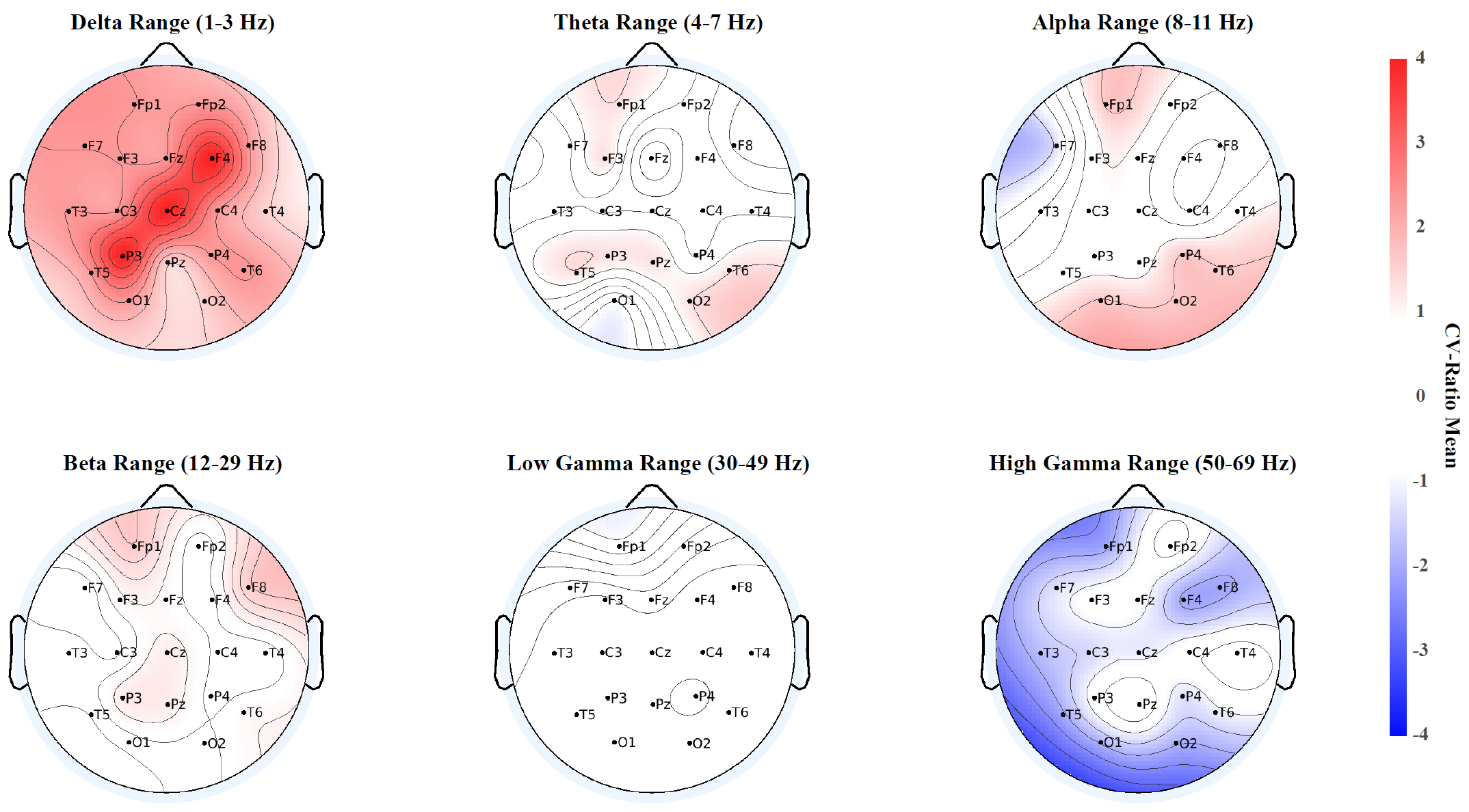
**

**Figure S6: Topographical Plots of Overall Mean of Cross-Validation Ratio in the EphysAGE Mode****l**

**Supplementary References**

1. Dukart J, Schroeter ML, Mueller K, The Alzheimer’s Disease Neuroimaging Initiative. Age Correction in Dementia – Matching to a Healthy Brain. Valdes-Sosa PA, ed. *PLoS ONE*. 2011;6(7):e22193. doi:10.1371/journal.pone.0022193

2. Koutsouleris N, Meisenzahl EM, Borgwardt S, et al. Individualized differential diagnosis of schizophrenia and mood disorders using neuroanatomical biomarkers. *Brain*. 2015;138(7):2059-2073. doi:10.1093/brain/awv111

3. St»hle L, Wold S. Analysis of variance (ANOVA). *Chemom Intell Lab Syst*. 1989;6(4):259-272. doi:10.1016/0169-7439(89)80095-4

4. Bonacich P. Some unique properties of eigenvector centrality. *Soc Netw*. 2007;29(4):555-564. doi:10.1016/j.socnet.2007.04.002

5. Benjamini Y, Hochberg Y. Controlling the False Discovery Rate: A Practical and Powerful Approach to Multiple Testing. *J R Stat Soc Ser B Stat Methodol*. 1995;57(1):289-300. doi:10.1111/j.2517-6161.1995.tb02031.x

6. Barry RJ, Clarke AR, Johnstone SJ, Magee CA, Rushby JA. EEG differences between eyes-closed and eyes-open resting conditions. *Clin Neurophysiol*. 2007;118(12):2765-2773. doi:10.1016/j.clinph.2007.07.028

7. Barry RJ, De Blasio FM. EEG differences between eyes-closed and eyes-open resting remain in healthy ageing. *Biol Psychol*. 2017;129:293-304. doi:10.1016/j.biopsycho.2017.09.010
